# Gender differences in time use across age groups: A study of ten industrialized countries, 2005–2015

**DOI:** 10.1371/journal.pone.0264411

**Published:** 2022-03-09

**Authors:** Joan García Román, Pablo Gracia

**Affiliations:** 1 Centre d’Estudis Demogràfics, Bellaterra, Spain; 2 Trinity College, Dublin, Ireland; Istituto di Ricerche Farmacologiche Mario Negri, ITALY

## Abstract

This study uses largescale cross-national time-diary data from the Multinational Time Use Study (MTUS) (N = 201,972) covering the period from 2005 to 2015 to examine gender differences in time use by age groups. The study compares ten industrialized countries across Asia, Europe, and North America. In all ten countries, gender differences in time use are smaller in personal care, sleeping and meals, followed by leisure time (including screen-based leisure and active leisure), and largest in housework, care work and paid work activities. Gender disparities in time use are higher in South Korea, Hungary, and Italy, followed closely by Spain, with moderate gender gaps in Western European countries like France and Netherlands, and lowest differences in Finland and Anglo-Saxon countries, including Canada, US, and the UK. Gender differences in housework and caring time increase from adolescence (10–17 years) to early adulthood (18–29 years), showing strong gender gaps in early/middle adulthood (30–44 years), but narrow again during late adulthood (65 years or older). However, the age gradient in care work and housework is most pronounced in Italy and South Korea, being less prominent in Canada and Finland. Gender gaps in paid work are larger in early/middle adulthood (30–44) and middle/late adulthood (45–64), with strongest age gradients observed in the Netherlands and weaker gradients for the US. Gender differences in active leisure increase by age, especially in Southern European countries, while screen-based leisure shows more stable gender gaps by age groups across different countries. Overall, this study shows that age and gender intersect strongly in affecting time-use patterns, but also that the national context plays an important role in shaping gender-age interactions in time use allocation.

## Introduction

The last decades have seen remarkable progress towards gender equality in how men and women spend time in multiple activities. Since the 1970s women have entered massively into the labor force, while men have significantly increased their participation in domestic tasks, especially in childcare activities [1]. However, research reveals that women remain more actively involved than men in housework and childcare, and less active in employment, leading to women’s disadvantages in income, health, and stress levels [2, 3]. Previous studies found that gender gaps in time spent on paid work and domestic chores augment during early adulthood and early/middle adulthood, which are life stages when partnership and parenthood transitions are most common [4–9]. While gender differences in time use across parenthood transitions have been well documented, the role of national contexts in shaping gender differences in daily activities across the whole life course (e.g., by age groups) remains poorly understood.

To our knowledge, only one study has systematically examined gender gaps in time use across age groups in cross-national perspective [10]. Using 1998–2004 time-diary data from individuals aged 18 or older in some selected Western European countries and the United States, Anxo et al. (2011) [10] found that countries with more active gender egalitarian policies and norms (i.e. Sweden) are more effective at reducing motherhood penalties in (un)paid work time during early and mid-adulthood, compared to countries with more gender traditional contexts (i.e., Italy). This study implies that national contexts can play a central role in shaping gender-age interactions associated with time-use patterns. While the mentioned study makes a relevant contribution, it only includes data from Western Europe and the US and uses surveys restricted to the late 1990s and early 2000s and excluding non-adults (i.e., children, teenagers) from the analyses. Also, the study by Anxo and colleagues [10] focuses only on three broad activities (employment, domestic work, and leisure in general), missing a rich variety of important activities to understand individuals’ well-being, including not only a differentiation between housework and care work, but also other key activities for individuals’ well-being and health like exercising, sleeping, eating or intellectual activities. These gaps motivate new research using more recent time-use data, adding previously excluded countries and regions, and focusing on multiple activities from childhood to late adulthood.

Our study contributes to the literature with a new cross-national analysis of gender differences in time-use allocation across age groups. To do so, we examine high-quality harmonized time-diary data from 2005 to 2015 across ten industrialized countries encompassing Asia, Europe, and North America. Drawing on the gender regimes literature, we hold that countries with different cultural, ideological and policy characteristics create different conditions for the development of power relations between men and women [11, 12] We argue that countries with different gender regime types may differ in the relative opportunities that society offers to men and women to use their time across age groups. Countries with stronger gender egalitarian policies and norms exhibit high levels of gender symmetry in activities like paid work, domestic labor, and leisure [5, 13–18]. Additionally, gender differences in time use may be less subject to age-specific stages from early to late adulthood in more gender egalitarian countries (i.e. Nordic countries like Finland; Anglo-Saxon countries like Canada), where family policies and gender norms provide support to both men and women to engage in similar time-use arrangements in those ages with high family and caring demands, namely early/middle adulthood, when parenthood transitions are more extended [15, 16]. By contrast, industrialized regions that are more gender unequal in their polices and norms, such as Southern Europe (i.e., Italy), East-Central Europe (i.e. Hungary) and East Asia (i.e., South Korea), may show particularly pervasive gender disparities in paid work, leisure or domestic work during early/middle adulthood, as these countries often have weaker institutions and social support to promote gender equal time-use arrangements targeting this demographic group.

Our cross-national study bridges the sociological and demographic literatures on time-use and life-course by analyzing gender differences in individuals’ time use across age groups that range from childhood to late adulthood. Drawing on the life course perspective, which emphasizes the importance of time, context, process and meaning in individuals’ development over the years [19], we analyze gender disparities in time use across five relevant age groups that capture distinct life-course stages in individuals’ biographies which have been similarly applied in the human development and demographic literatures [20, 21]: (1) adolescence (ages 10–17); (2) early adulthood (ages 18–29); (3) early/middle adulthood (ages 30–44); (4) middle/late adulthood (ages 45–64); (5) late adulthood (aged 65 or older). Examining in what national contexts men and women adopt more (or less) similar time-use arrangement by ages helps to better understand the individual and societal dynamics of gender differences in lifestyles and daily routines across the life span. For reasons of space and focus, our study does not concentrate on other important indicators of the life course, such as being a parent versus being childless, living with parents versus having left home already and being partnered compared to being single. However, we conduct analyses that intersect age and gender which control for important markers of the life course, such as family structure and number of children. Age is a crucial life-course marker to study individuals’ time-use allocation. Age is a measure that allows us to assess how a universally comparable biological marker relates to different gendered patterns in time use allocation, but also whether these gender-age interactions are more or less similar or stable depending on the national context.

Our study makes three main contributions to the international literature on gender differences in time use by considering both the *absolute* (total time) and *relative* (percentages) gender gaps across all countries of study. First, unlike previous studies, we use the most recent data available from the Multinational Time Use study (MTUS) between 2005 and 2015 for ten countries, encompassing regions that have been omitted in previous comparative literature, such as East-Central Europe (i.e., Hungary), and East Asia (i.e., South Korea), besides Western Europe, North America, and Southern Europe. Second, unlike previous studies, we examine time-use patterns from adolescence (10–17 years old) to old age (65 years old and older). Third, unlike previous studies, we not only study paid work, housework, care work and leisure in general, but also focus on other key markers of men’s and women’s health and well-being over their life course: sleeping, personal care, eating and studying. Previous research has found that lacking enough sleeping time is associated with poorer health outcomes and mental well-being [22–26]. Studies also found that time spent in physical and active leisure helps to prevent pathologies and mental health problems, while more sedentary leisure–particularly high levels of screen-based leisure–has been found to be associated with physical and mental health problems, especially among the younger cohorts [24, 27, 28]. Overall, our study adds to the scarce literature on gender gaps in time use across age groups by adding a cross-national approach on multiple activities with different well-being implications.

Globally, this study demonstrates the importance of national context in shaping age-gender interactions that define individuals’ time-use patterns over the life course. Our study represents a clear example of how using precise time-diary data provides unique understandings of the role of societal contexts in shaping how age impacts the way men and women engage in activities that are essential to understand differences in health, well-being or income across countries. These time-use differences are crucial to inform debates on gender inequalities across policy and cultural contexts.

## Data and methods

We use data from the Multinational Time Use Study (MTUS), the largest openly accessible nationally representative harmonized time diary survey database that provides data comparable across time and space [29]. Time-diary data are considered the most precise and robust statistical sources available to measure individuals’ behavior [e.g., 18, 30]. Time-use diary surveys provide nationally representative samples of comprehensive, continuously-registered, records about the activities of daily life [31]. The data were obtained from the MTUS-X extract system (www.mtusdata.org), allowing us to personalize the selection of samples and surveys [32]. We obtained large diary data collected between 2005 and 2015 from ten industrialized countries representing different gender regimes (N = 201,972). S1 Appendix shows a summary of our sample. The selection of countries is both strategic and practical. From a strategic point of view, we are interested in countries that have different social, cultural, and policy environments to explore the macro-level determinants of gender differences in time use. From a practical point of view, we are limited by data access. Not all countries that carried out time use surveys are available in the MTUS and access to multiple surveys is either restricted or challenging in terms of data harmonization. Drawing on previous cross-national frameworks on gender attitudes and work-family policies [e.g., 13, 17, 18], our ten countries can be integrated into six clusters:

- *Anglo-Saxon*: Canada (2010), United Kingdom (2014), United States (2010)
- *East Asia*: South Korea (2009)
- *East-Central Europe*: Hungary (2009)
- *Nordic Europe*: Finland (2009)
- *Southern Europe*: Spain (2009), Italy (2008)
- *Western Europe*: France (2009), Netherlands (2005)

We created twelve groups of activities across a random 24-hours day from the time diaries, adding the average number of daily minutes devoted to each activity. The aggregated variables are created according to the main activity reported in the diary. The list of activities and the main activities and codes considered in each group are as follows:

- Sleeping (codes 2–3): Sleeping, naps, imputed sleep.
- Personal care (codes 1 and 4): Washing up, dress, care for self, imputed personal.
- Meals (codes 5–6): Meals, snacks.
- Paid work (codes 7–9, 11–13): paid work at main or second job, travel as part of work, work breaks.
- Study (codes 15–17): regular schooling, homework, leisure or other education or training.
- Housework (codes 18–27): food preparation, cooking, setting the table, washing dishes, cleaning, laundry, ironing, clothing repairs, maintaining home or vehicle purchase goods, consuming personal care services, pet care.
- Care for others (codes 28–32): physical and medical childcare, teach and help child with homework, reading to, talking or playing with child, supervise, accompany a child, adult care. We include all types of activities of care for others within one category. We acknowledge that each type of care (e.g., care for adults versus care for children) has a different nature and implications for the person who does the activity. We decided to integrate all care as a single activity for reasons of space, but also because the average minutes allocated to caring for adults and the proportion of respondents who engaged in this activity is low.
- Active leisure (codes 42–47): general sport or exercise, walking or cycling, other outside recreation, walking with pets.
- Screen-based leisure (codes 59–61): watch TV, video or dvd, computer games, e-mail, surfing the internet or computing.
- Other Leisure (codes 33–41 and 48–58): voluntary activities, worship and religion, general out-of-home leisure, attend sports events, cinema theatre, opera, concerts, other public events, restaurant, cafe, bar, pub, party, social event, gambling, receive or visit friends, conversations, games, general indoor leisure, art or music, correspondence, knit, crafts or hobbies, relax, think, read, listen to music or radio. We include social and cultural activities as well as those leisure activities that are not specified enough to be classified in the more detail leisure categories.
- Travel (codes 62–68): travel to/from work, voluntary travel, child/adult care travel, other travel
- Other activities (code 10, 14, 69):

We have excluded time in travel and other activities from the more detailed analyses of our study. There were not significant differences in these activities across countries throughout a full random day of 24 hours (1440 minutes).

Our analyses focus on five age groups that, as explained above, generally correspond to meaningful stages of the life course across industrialized countries, which have been previously associated with gendered life-course stages over the life course:

- Age: 10–17 (For Canada and the United States, includes the ages of 15–17, as there are no data available for younger individuals), 18–29, 30–44, 45–64, 65 and older

We present general average measures by age, gender and country. In additional analyses (not shown) we have computed multivariate OLS models controlled for basic demographics (i.e. education, employment status). Results were generally in line with our main findings. We decided to take out these controls from the model, which partly condition our estimations. For example, countries differ in female employment policies, which affect gender differences in employment status, and this in turn contributes to explain the gender gap in time use). In the descriptive analysis, first, we show the general average time (daily minutes) allocated to each activity over the day (1440 minutes). Second, we show the average time for each activity by country and age group. Third, we explore gender gaps in each activity of interest by using the following formula of time-use gender ratio [16, 33]:

$$Gender\;relative\;ratio=\frac{Men\prime s\;Time-Women\prime s\;Time}{Men\prime s\;Time+Women\prime s\;Time}$$

The time-use gender ratio shows differences between men and women in time use. It goes from -1 (when women do all time in the specific activity) to 1 (when men do all time). Positive values mean that men spend more time in the activity, with negative values indicating that women spend more time in the activity. The value 0 indicates no gender differences in time use. Averages correspond to the average by week including weekends and weekdays. To compute averages, we have used the proposed weights (PROPWT) provided by the MTUS to rescale the population and day of the week. Applying weights is necessary in time use surveys when weekdays and weekend diaries are integrated within the same analyses. Weights correct for the overrepresentation of weekends respondents in some surveys and imbalances in the population’s structure by age, gender and employment status. There are not missing values in the harmonized variables used in our analysis, although there might be some imputation in the procedures to create the harmonized files that the MTUS-X provides.

Finally, we conduct multivariate analyses to estimate gender differences in the time spent on each activity within country and age groups. Using Ordinary Least Squares (OLS) regression, we run 500 models (10 activities * 10 countries * 5 groups of age = 500 regressions) so that each coefficient (500 in total) presents the differences between men and women in estimating activity X within the same country and age group. We are not running Compositional Data Analysis, as we are not combining multiple activities within the same statistical model, but rather estimate each activity separately [34–38]. Previous studies show that OLS regressions are robust estimation techniques for cross-sectional observational time-diary data and a better alternative than Tobit regressions [39]. In these final analyses, we start by presenting the coefficient and significance test of gender on time use for each group and country, after controlling for day of the week. Subsequently, we reproduce the analyses of the effect of gender on time use, adjusting for socioeconomic and demographic factors in each model. We are limited by the harmonized variables in the original dataset, especially for the group 10–17, but we can use the following sociodemographic controls:

- Educational attainment: respondent is under18 or value is missing, less than secondary, completed secondary, above secondary
- Employment status: employed or not employed
- Couple status: in union or not
- Children status: respondent is under 18, without coresident children in the household, coresident children under 5 years old in the household, coresident children 5–17 household. Take into account that we refer to coresident children who may not be the respondent’s children.

Finally, we run several tests to check the necessary assumptions to conduct OLS regressions, including checks of homoscedasticity and multicollinearity in all OLS regressions. Analyses suggest that parameters to run OLS regressions are correct. For all models obtained, we run tests in Stata, using vif and imtest commands. Moreover, the number of observations in all regressions is large enough to assume normality in the data (see S1 Appendix to check our sample sizes).

## Results

### Description of gender differences in time use in ten countries

**Fig 1:**
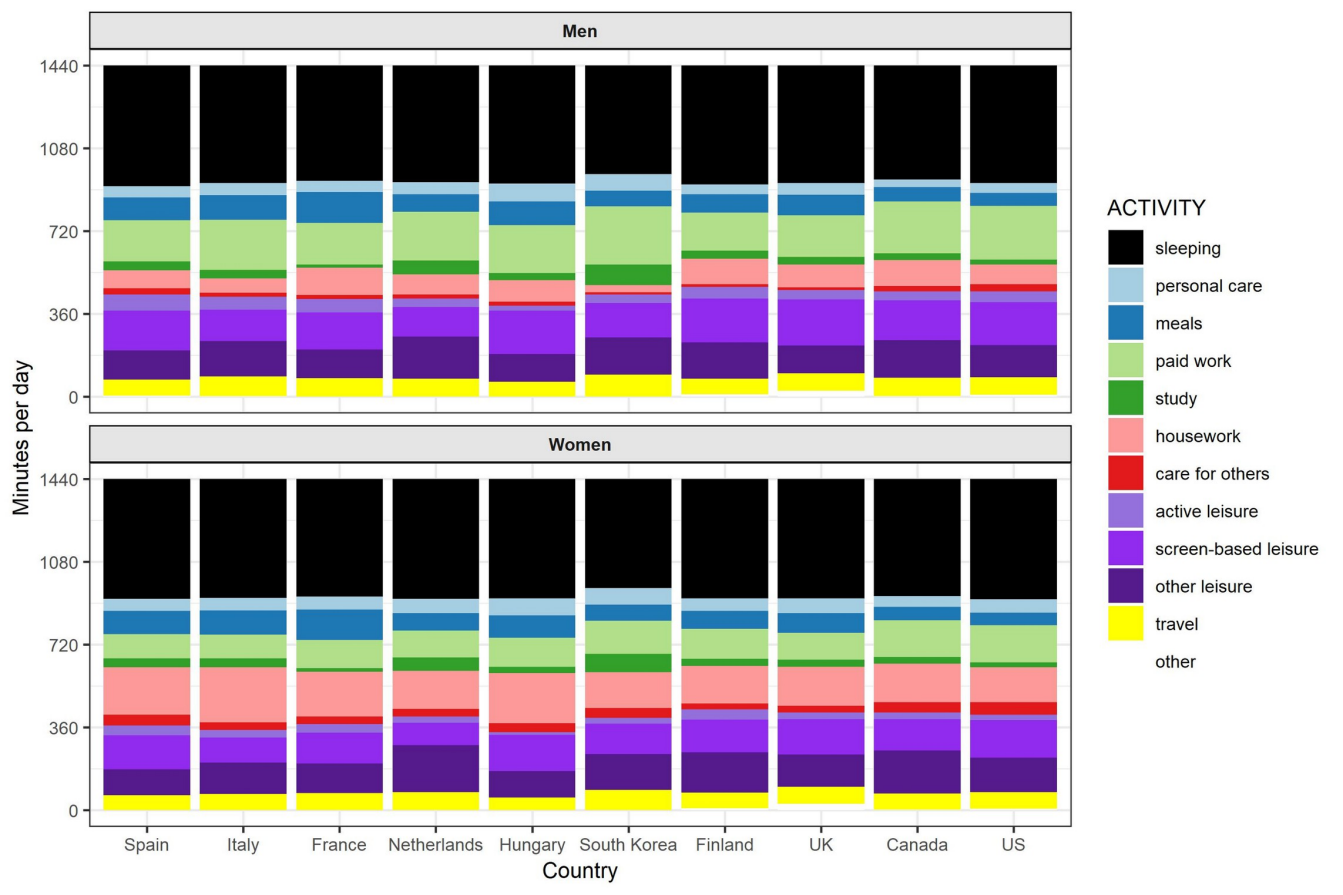
Average time by activity. Men and Women by country. Source: Own calculations from the Multinational Time Use Study [32]. *Activities created from the variable *Main activity*: Sleeping (codes 2 and 3), personal care (1 and 4), meals (5 and 6), paid work (7 to 13, except 10), study (15 to 17), housework (18 to 27), care for others (28 to 32), active leisure (42–47), screen-based leisure (59 to 61), other leisure (33–41 and 48–58) (see methods section). Other two activities include: travel (codes 62 to 68), others (10, 14 and 69). **Estimates of the figure available in Table 1.

Fig 1 and Table 1 present the average daily minutes in each activity by country, separately for men and women. The total time is 1440 minutes, that is 24 hours. Estimates correspond to the average time of the overall population in each country. We only report differences for our ten activities of study, without discussing differences in travelling and ‘others.’

**Table 1:** Average time and standard deviation by activity. Men’s and Women’s daily minutes by country.

SEX	ACTIVITY	Spain	Italy	France	Netherlands	Hungary	South Korea	Finland	UK	Canada	US
Men	Sleeping	524	501	513	517	496	473	517	512	496	510
		(124.9)	(107.7)	(118)	(117.8)	(121.8)	(96.7)	(117.8)	(129)	(121.8)	(134.2)
	Personal care	49	49	79	43	33	71	43	51	33	43
		(32)	(38.3)	(52.2)	(36.5)	(37.8)	(31.4)	(36.5)	(40.1)	(37.8)	(57.7)
	Meals	100	136	103	79	62	69	79	89	62	58
		(47.6)	(73.9)	(52.8)	(44.3)	(57)	(46.9)	(44.3)	(64.9)	(57)	(50.3)
	Paid work	179	181	207	166	225	252	166	181	225	234
		(254.8)	(244.3)	(247.5)	(245.4)	(268.2)	(248.3)	(245.4)	(248.3)	(268.2)	(269.4)
	Study	39	13	31	35	29	90	35	33	29	21
		(122)	(74.4)	(107.1)	(111.5)	(107.5)	(197.1)	(111.5)	(108.8)	(107.5)	(92.3)
	Housework	78	118	95	110	115	31	110	99	115	86
		(99.2)	(124.7)	(105.7)	(116.6)	(138.6)	(55.1)	(116.6)	(107.6)	(138.6)	(117.5)
	Care for others	27	19	17	11	23	10	11	13	23	30
		(72.2)	(57.8)	(49.4)	(41.9)	(73.3)	(35.3)	(41.9)	(43.1)	(73.3)	(76.8)
	Active leisure	69	57	21	51	39	36	51	41	39	47
		(96.5)	(95.2)	(58.7)	(81.1)	(83.6)	(64.8)	(81.1)	(72.9)	(83.6)	(96.1)
	Screen-based leisure	173	163	189	190	172	151	190	199	172	188
		(140.1)	(137.4)	(136.1)	(148.5)	(167.1)	(132.8)	(148.5)	(164.3)	(167.1)	(180.5)
	Other leisure	127	123	120	158	165	160	158	121	165	140
		(133.6)	(132.1)	(121)	(147.8)	(180.6)	(104.5)	(147.8)	(134.8)	(180.6)	(162.9)
	Travel	72	79	65	68	79	95	68	77	79	77
		(66)	(83)	(68.5)	(82.1)	(83.6)	(69.2)	(82.1)	(80)	(83.6)	(79.9)
	Other	4	1	0	11	3	2	11	25	3	8
		(32)	(7.6)	(1.1)	(58.1)	(28.5)	(16.7)	(58.1)	(99.7)	(28.5)	(39.8)
Women	Sleeping	521	511	519	519	510	569	635	641	617	644
		(114.4)	(107.1)	(100.8)	(106.4)	(120.6)	(101.9)	(79.7)	(90.7)	(71)	(100.8)
	Personal care	53	57	75	54	46	116	123	154	119	108
		(34.6)	(39.3)	(52.7)	(36.8)	(40.1)	(500.1)	(411.5)	(429.2)	(493.4)	(503.1)
	Meals	100	132	97	79	58	287	146	142	136	113
		(45.9)	(70.6)	(46.1)	(43.3)	(55)	(86)	(227.1)	(206.9)	(253.2)	(203.6)
	Paid work	106	122	126	130	159	46	53	56	96	107
		(193.4)	(203.6)	(197.1)	(209.7)	(231.7)	(100.6)	(132.1)	(113.6)	(122.5)	(142.9)
	Study	37	16	28	32	29	284	338	364	339	368
		(119.9)	(81.7)	(102.3)	(101.7)	(107.7)	(264.8)	(305.5)	(255.6)	(345.3)	(302.5)
	Housework	207	195	218	163	169	165	150	157	163	156
		(151.5)	(134.7)	(139.8)	(123.6)	(148.7)	(18.3)	(69.4)	(125)	(31.6)	(47.7)
	Care for others	47	33	39	24	44	474	519	520	510	524
		(95.5)	(71.9)	(89.3)	(73.3)	(102.6)	(94.3)	(106.4)	(123.7)	(120.6)	(132)
	Active leisure	44	35	11	45	29	72	54	64	46	57
		(68.3)	(64.6)	(38.4)	(66.4)	(66.7)	(38.2)	(36.8)	(45)	(40.1)	(64.7)
	Screen-based leisure	147	136	157	143	136	71	79	86	58	54
		(121.4)	(122.6)	(115.1)	(116.9)	(140.7)	(41.9)	(43.3)	(61.3)	(55)	(47.5)
	Other leisure	113	128	116	174	186	143	130	116	159	163
		(122.6)	(125)	(113.9)	(139)	(179.9)	(208.5)	(209.7)	(203.1)	(231.7)	(235.7)
	Travel	64	73	55	68	71	81	32	32	29	20
		(61.4)	(77.7)	(60.2)	(80.2)	(77.1)	(187.4)	(101.7)	(104.7)	(107.7)	(89.5)
	Other	1	1	0	9	2	155	163	169	169	152
		(16.2)	(7.7)	(1.1)	(41.2)	(22.5)	(124.7)	(123.6)	(130)	(148.7)	(142.2)

Source: Own calculations from the Multinational Time Use Study [32].

Fig 1 shows that men spent more time than women on paid work and also, even with smaller gender differences, on leisure activities, particularly in relation to active and screen-based leisure. Women allocated more time to housework, caring and–to a lesser extent–personal care. Patterns in sleeping time, study and meals were similar by gender. More important, the magnitude of gender gaps in time use differed remarkably across countries (see Table 2).

**Table 2:** Gender gap measure by activity and country.

Measure	ACTIVITY	Spain	Italy	France	Netherlands	Hungary	South Korea	Finland	UK	Canada	US
Gender gap (difference)	Sleeping	3	-7	-11	-14	-6	-1	-2	-8	-14	-14
	Personal care	-5	-1	-8	-8	4	-2	-11	-14	-12	-14
	Meals	-1	0	4	0	6	-2	0	3	4	3
	Paid work	73	116	59	94	81	108	37	65	66	71
	Study	2	-2	-3	3	4	10	3	1	-1	1
	Housework	-129	-176	-78	-77	-123	-124	-52	-69	-54	-66
	Care for others	-20	-17	-14	-18	-22	-31	-13	-17	-21	-24
	Active leisure	26	25	22	9	9	9	5	12	10	22
	Screen-based leisure	26	26	27	30	32	19	47	46	36	25
	Other leisure	14	17	-5	-19	5	5	-17	-20	-22	-10
	Travel	8	17	7	-1	10	8	0	5	8	4
	Other	3	1	0	0	0	1	2	-3	1	2
Gender gap (indicator)	Sleeping	0.003	-0.007	-0.011	-0.014	-0.006	-0.001	-0.002	-0.008	-0.014	-0.013
	Personal care	-0.045	-0.006	-0.079	-0.074	0.025	-0.013	-0.114	-0.118	-0.158	-0.141
	Meals	-0.003	0.002	0.015	-0.002	0.030	-0.012	0.001	0.015	0.030	0.029
	Paid work	0.257	0.363	0.195	0.288	0.243	0.275	0.124	0.218	0.172	0.179
	Study	0.027	-0.024	-0.102	0.028	0.063	0.056	0.044	0.020	-0.010	0.014
	Housework	-0.454	-0.579	-0.249	-0.306	-0.394	-0.668	-0.192	-0.259	-0.191	-0.277
	Care for others	-0.268	-0.338	-0.263	-0.348	-0.401	-0.601	-0.362	-0.410	-0.322	-0.284
	Active leisure	0.227	0.275	0.233	0.146	0.292	0.153	0.057	0.167	0.139	0.311
	Screen-based leisure	0.081	0.107	0.091	0.134	0.094	0.066	0.141	0.129	0.118	0.070
	Other leisure	0.057	0.060	-0.019	-0.049	0.019	0.015	-0.050	-0.075	-0.061	-0.034
	Travel	0.057	0.107	0.044	-0.004	0.087	0.045	0.003	0.031	0.052	0.026
	Other	0.516	0.598	-0.034	0.145	-0.028	0.620	0.123	-0.055	0.235	0.161

Source: Own calculations from the Multinational Time Use Study [32].

*Note*: For other, the point indicates that the value cannot be computed mathematically.

In Fig 1, we observe small gender differences in *sleeping time*. Sleeping takes between 8 and 9 hours, with a maximum of 8 hours and 45 minutes for women in Italy (one minute more than Spanish men and US women) and a minimum of 7 hours and 54 minutes for both genders in South Korea. Time in *personal care* differed remarkably across countries, ranging from 79 minutes for Hungarian men (75 for women) to 33 minutes for Canadian men (46 for women). Gender differences in personal care seem slightly higher in Anglo-Saxon countries and Finland (women spend around 1.4 and 1.3 times more than men, respectively) than in the other countries. For *meals*, French, Spaniards, and Italians spent the highest amount of time (about 2 hours 15 minutes in France, 1 hours 45 minutes in Italy and 1 hour and 40 minutes in Spain), and Americans, Canadians, and the Dutch the least. However, gender differences in eating time were generally small in all countries.

Fig 1 shows clear gender gaps in paid work time and domestic work across countries. For *paid work*, the most active men were South Koreans (253 minutes) and the least active ones were the Finnish (166 minutes). For women, Americans and Canadians were the most involved in paid work, with 165 and 161 minutes respectively, and Italians the least (95 minutes). Finland, France, Canada, and US had the lowest gender gaps in employment activities, and Italy, Spain and South Korea showed the largest paid work gender gaps.

As for *housework*, men in South Korea (31 minutes) were the least active and men in France (118 minutes) and Canada (115 minutes) the most active (see Fig 1). For women, the Italian spent the highest amount of time in housework (226 minutes) and South Koreans and Americans the lowest (155 minutes and 152 minutes, respectively). Gender differences in housework were largest in Southern Europe, Hungary, and South Korea and lowest in Anglo-Saxon countries and Finland. Regarding *care time*, American women showed the highest average time, whereas South Korean presented the lowest involvement in care work (10 minutes). Gender gaps in time allocated to caring activities were highest in South Korea and smallest in Finland.

For *leisure* activities, we observed the highest averages of time for screen-based activities and the lowest in active leisure. For screen-based leisure, the highest averages were observed among males in UK (199 minutes) followed closely by males in Finland, Hungary and the US (between 190 and 188 all). Among females, the US shows the highest average minutes allocated to screen-based activities (163 minutes). Time on screen-based leisure is lowest among the Dutch (128 minutes for males and 98 minutes for females) (see Fig 1). For active leisure we found the highest averages in Spain (69 minutes for males and 44 for females, respectively) and the lowest among Hungarians (21 minutes for males and 11 for females). While gender differences in screen-based time were largest in Finland, Netherlands and the UK (and lowest in South Korea and the US), gender gaps in active leisure were largest in the US (and smallest in Finland).

*Study* time showed values close to zero, as most adults did not engage in these activities. South Korea was by far where people spent more time of study (1 hour and 30 minutes for men; 1 hour and 21 minutes for women). The lowest means of study time were found for France and the United States, with a minimum of 13 minutes for French men.

### Unadjusted relative gender gaps in time use by age groups and countries

**Fig 2:**
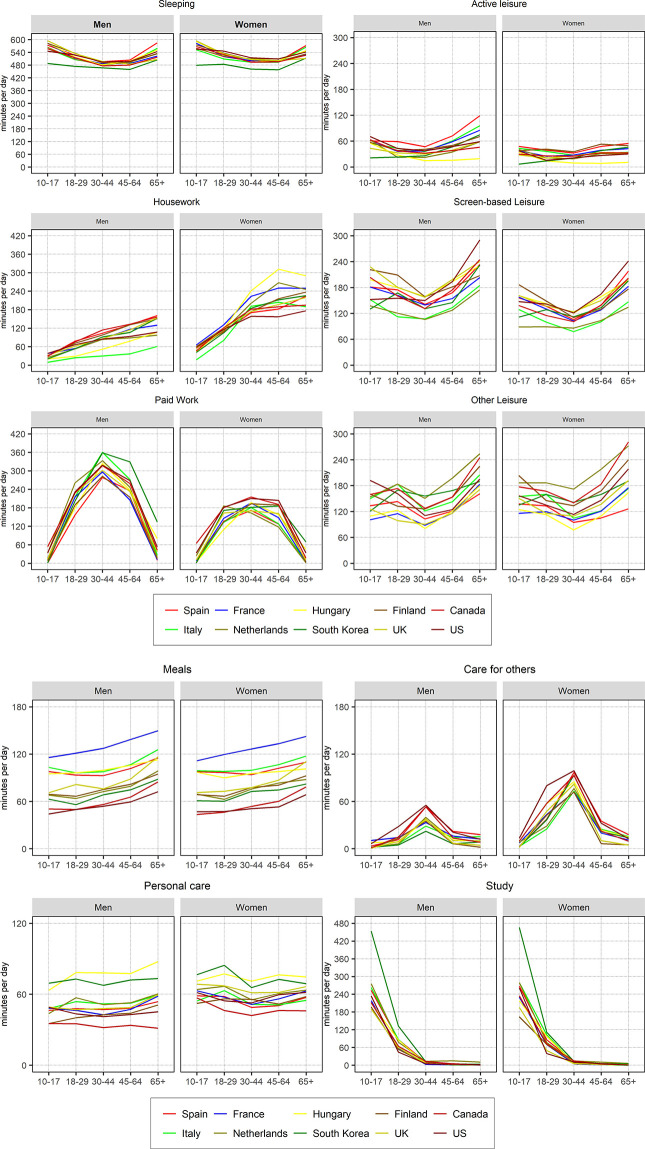
Average time by activity and age. Men and women by country. Source: Own calculations from the Multinational Time Use Study [32]. *Estimates of the figure available in Table 3.

Fig 2 and Table 3 present the average time spent by men and women in each activity by age groups across countries. While the direction of patterns by age groups were quite similar across all ten countries, the magnitude of these gendered gaps differed cross-nationally. In Table A2 in S1 Appendix we present the percentage of people who have participated in each activity by age, gender and country. There are some activities such as *sleeping*, *personal care* and *meals* for which the average time was generally flat by age and gender, even if time on meals tended to increase for the older demographic groups (i.e., 45–64; 65 or older). For *studying*, not surprisingly, the highest averages appeared in the 10–17 age group, followed by the 18–29 group, while afterward the average study time was almost zero for both men and women. Participation rate in studying activities in adult population was very low and, as a result, the average was low too, as visible in Table A2 in S1 Appendix. As mentioned, South Korea was an outlier in study time, with very high average minutes among both boys and girls. Time in personal care differs between countries, as mentioned already, but we observe an interesting stability within countries when comparing personal care across age groups.

**Table 3:** Average time by activity and age. Men’s and women’s daily minutes by country.

		Spain		Italy		France		Netherlands		Hungary		South Korea		Finland		UK		Canada		US	
AGE	ACTIVITY	M	W	M	W	M	W	M	W	M	W	M	W	M	W	M	W	M	W	M	W
10–17	Sleeping	573	560	558	553	583	583	564	557	584	592	488	480	583	575	594	594	556	567	545	555
	Personal care	46	59	48	54	48	63	44	64	63	71	69	77	35	52	49	69	35	57	49	61
	Meals	98	98	103	99	116	112	68	69	95	97	63	61	69	69	71	71	50	43	44	47
	Paid work	2	2	9	3	16	7	33	24	14	4	1	1	9	11	15	11	54	65	33	34
	Study	254	267	262	279	218	230	276	279	210	218	453	466	197	164	190	195	212	235	234	262
	Housework	25	47	20	43	30	61	20	41	38	65	9	17	29	54	32	55	30	46	38	58
	Care for others	3	3	2	3	11	7	2	8	8	10	2	3	0	3	1	2	1	10	6	13
	Active leisure	61	48	55	43	62	38	63	41	55	34	21	6	57	38	43	28	64	30	70	37
	Screen-based leisure	181	156	152	129	181	158	138	88	198	161	130	111	221	187	228	162	204	138	152	147
	Other leisure	133	138	150	155	101	116	154	186	109	124	121	135	160	203	126	150	160	177	192	157
18–29	Sleeping	526	520	506	509	512	522	507	523	517	530	474	484	536	536	534	533	515	529	528	547
	Personal care	48	58	54	63	46	57	57	67	78	77	73	84	40	56	47	67	35	46	43	54
	Meals	93	97	96	98	121	120	64	63	96	90	56	60	67	67	82	73	50	46	50	47
	Paid work	157	137	216	133	215	146	261	186	180	110	202	172	190	135	228	163	229	180	232	182
	Study	86	86	86	103	55	70	77	77	85	98	132	112	54	70	60	50	64	72	44	39
	Housework	53	112	29	118	71	120	53	105	54	131	24	80	78	109	61	119	75	121	66	116
	Care for others	12	37	6	25	14	43	6	29	8	57	5	37	13	43	9	48	15	58	28	80
	Active leisure	59	39	43	36	36	25	23	21	27	14	23	14	38	42	33	21	37	26	43	15
	Screen-based leisure	174	137	112	100	162	130	120	89	180	143	168	128	209	149	181	137	160	115	155	142
	Other leisure	143	133	183	160	115	120	184	186	123	113	170	160	132	145	99	116	174	167	162	135
30–44	Sleeping	496	500	487	494	486	500	481	509	482	494	467	461	491	505	498	506	476	494	495	514
	Personal care	47	49	52	52	43	52	51	55	78	71	68	66	43	56	48	61	32	42	41	53
	Meals	93	94	98	99	127	127	72	76	99	95	68	73	75	78	76	78	56	54	54	51
	Paid work	278	172	360	181	297	196	333	163	324	197	359	182	283	193	302	170	317	215	318	210
	Study	10	13	3	5	3	4	13	14	6	6	12	13	6	8	6	5	13	14	5	11
	Housework	84	201	52	241	98	174	91	185	85	223	29	191	103	171	85	174	114	182	85	159
	Care for others	53	97	29	73	33	72	40	76	37	85	22	93	35	72	38	82	53	93	55	99
	Active leisure	47	33	36	27	37	28	22	19	15	9	26	21	41	36	30	26	32	25	38	21
	Screen-based leisure	141	105	107	78	139	102	106	86	159	121	131	112	159	110	157	124	131	101	150	121
	Other leisure	103	95	122	105	88	100	151	172	81	77	156	141	126	133	90	109	127	140	111	113
45–64	Sleeping	504	497	491	494	488	499	494	510	499	501	459	458	496	497	485	502	480	496	497	509
	Personal care	48	50	52	51	47	56	53	61	78	76	72	73	44	52	49	62	34	46	43	60
	Meals	102	102	107	107	139	133	79	83	106	98	75	75	82	81	88	87	66	60	59	53
	Paid work	235	127	272	128	205	148	247	118	237	158	329	186	215	189	233	162	259	191	268	204
	Study	5	6	1	2	1	2	15	10	2	2	4	5	3	6	3	3	4	4	3	5
	Housework	88	268	78	311	131	216	106	212	116	250	37	204	131	182	114	197	133	189	93	157
	Care for others	22	35	14	25	16	20	11	22	11	24	6	21	6	6	7	10	14	22	21	32
	Active leisure	72	48	61	32	59	39	35	31	16	9	46	38	50	53	39	34	38	33	48	27
	Screen-based leisure	168	139	133	101	155	128	127	103	194	159	145	133	181	130	199	150	174	140	194	165
	Other leisure	121	105	143	122	116	121	197	218	121	110	169	160	153	168	116	137	154	183	125	146
65+	Sleeping	585	572	559	564	523	529	533	544	552	548	504	513	535	532	507	511	518	526	544	541
	Personal care	54	57	59	55	58	63	60	63	88	75	73	69	51	58	59	66	31	46	45	62
	Meals	115	110	126	117	150	143	99	88	113	101	88	82	95	93	117	111	85	78	72	69
	Paid work	10	4	26	5	12	5	19	1	80	45	134	70	15	3	37	10	43	16	53	33
	Study	1	2	1	0	1	2	11	6	1	1	3	5	2	1	1	2	1	1	2	0
	Housework	97	245	107	290	151	237	152	226	130	249	61	190	161	224	154	219	156	195	107	177
	Care for others	18	18	15	16	12	11	10	14	10	15	9	14	2	5	4	4	8	9	12	13
	Active leisure	119	55	96	34	85	43	58	31	19	11	75	46	71	49	59	33	46	33	59	30
	Screen-based leisure	233	218	185	147	203	183	175	134	231	196	232	196	208	176	240	200	245	202	290	242
	Other leisure	161	126	205	174	183	175	254	272	180	164	190	191	225	240	171	192	245	281	195	219

Source: Own calculations from the Multinational Time Use Study [32].

For *housework*, women spent more time than men at all ages. For men, housework time increased with age (see Fig 2). For women, housework augmented up to middle adulthood, and then the pattern differed cross-nationally. In Southern Europe and South Korea, with more gender traditional norms, women’s housework increased until the ages of 45–64 and decreased for the elderly. In Anglo-Saxon and Western European countries, women’s housework time moderately increased for the elderly. Hungary exhibited a certain stabilization of housework time in old age (45–64; 65+).

For *paid work*, we found an increase of time until the age group 30–44, a slight decrease in the 45–64 age group, and more pronounced declines among the group aged 65 and older (see Fig 2). For men, this pattern was common everywhere. Yet, in South Korea, a long-hours working regime, the average of paid work for the group of 65+ was above 2 hours, and in Hungary 1 hour and 20 minutes. In the US and Canada, where retirement is not strongly supported by the welfare state, men aged 65 or older spent 53 and 45 minutes in paid work, respectively. By contrast, in EU countries where people retire early, such as Spain, France and Finland, men in the eldest group spent less than 15 minutes in paid work. For women, we found important cross-national variations in women’s paid work across age groups. In some countries, women’s paid work for the 30–44 and 45–64 age groups were similar or even higher for the older group, as in South Korea, Finland and Anglo-Saxon countries. In the Netherlands, women aged 18–29 were the most active in paid work (186 minutes, one minute more than US women). However, Dutch women were the least active in paid work across all ten countries within the 30–44 age group (163 minutes). This pattern shows the institutionalization of short part-time employment among Dutch women in this age group.

For screen-based *leisure*, the age progression in time use showed a U-shape for both men and women, with a reduction of time for the 30–44 age group and largest amounts of time for the youngest and eldest groups (see Fig 2). The highest amounts of screen-based time were globally found in the group of age 65 and older, especially in the US. For active leisure, time-use patterns differed across countries. In the Mediterranean countries, averages were quite constant in both the youngest and middle-age groups and increased among older groups. In Anglo-Saxon countries, averages decreased for the adult age groups and went up among the late-adulthood groups, but not as much as in Mediterranean countries. South Korea shows the lowest averages for active leisure among the youngest groups, with a subsequent increase for the older groups, while Hungary shows the lowest averages across all age groups. Men spent more time than women in both active leisure and screen-based leisure in all countries and age groups.

Finally, regarding *caring activities*, the general pattern showed an inverted U-shape, but especially so among women (see Fig 2). The age group 30–44 was the most active in care activities in all countries. For the US, Women in the group 18–29 were very active in caring activities with an average of 80 minutes, rising to almost a hundred in the group 30–44. Men in Italy and South Korea remained little involved in care work, even during the 30–44 age groups, when care needs are highest. For the eldest group (65 or older), both Spanish men and women were the most active in care work, with men and women in Anglo-Saxon countries being the least involved in these activities (only 4 minutes for both genders).

### Unadjusted relative gender gaps in time use by age groups

**Fig 3:**
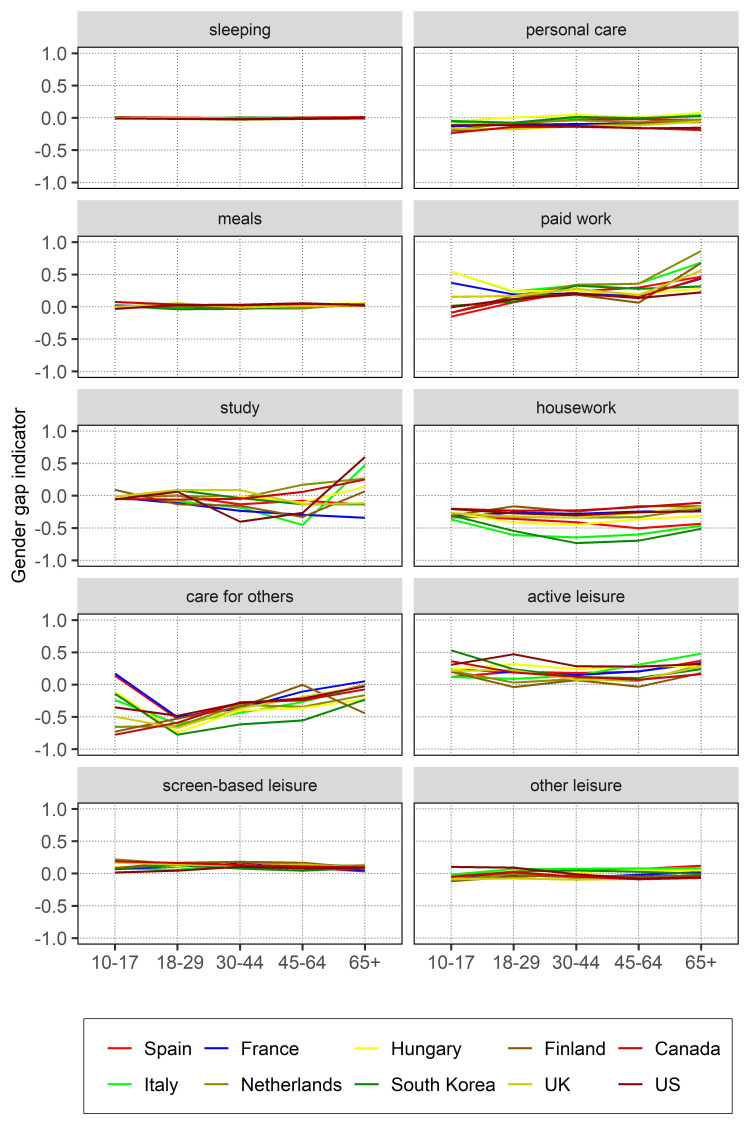
Gender gaps in time use (Indicator from -1 to 1). Differences in activities by age. Source: Own calculations from the Multinational Time Use Study [32]. *Estimates of the figure available in Table 4.

Fig 3 and Table 4 show the relative gender gaps in time use by activity and age across countries. We show a gender ratio indicator that ranges from -1 (women did all the time in the activity) to +1 (men did all the time in the activity). Relative gender gaps were strongest for domestic work, paid work and, to a lesser extent, leisure. For *screen-based leisure*, the gender gap indicator was almost flat across age groups, with men spending more time across the different groups of age. Gender gaps in active leisure were also positive and higher for children and the older population groups.

**Table 4:** Gender gaps in time use (Indicator from -1 to 1). Differences in activities by age.

AGE	ACTIVITY	Spain	Italy	France	Netherlands	Hungary	South Korea	Finland	UK	Canada	US
10–17	Sleeping	0.012	0.005	0.000	0.006	-0.007	0.008	0.007	0.001	-0.010	-0.009
	Personal care	-0.117	-0.063	-0.131	-0.189	-0.058	-0.050	-0.194	-0.165	-0.235	-0.112
	Meals	0.000	0.022	0.019	-0.009	-0.011	0.018	0.003	-0.001	0.075	-0.031
	Paid work	-0.151	0.545	0.371	0.154	0.546	0.014	-0.093	0.155	-0.090	-0.005
	Study	-0.026	-0.032	-0.027	-0.007	-0.019	-0.014	0.092	-0.014	-0.051	-0.056
	Housework	-0.310	-0.367	-0.331	-0.337	-0.262	-0.304	-0.307	-0.268	-0.204	-0.205
	Care for others	0.140	-0.238	0.171	-0.654	-0.110	-0.144	-0.729	-0.496	-0.777	-0.351
	Active leisure	0.118	0.119	0.242	0.214	0.236	0.531	0.198	0.215	0.363	0.306
	Screen-based leisure	0.074	0.082	0.069	0.218	0.103	0.077	0.085	0.171	0.191	0.016
	Other leisure	-0.016	-0.016	-0.069	-0.096	-0.064	-0.055	-0.119	-0.089	-0.052	0.102
18–29	Sleeping	0.006	-0.003	-0.009	-0.016	-0.012	-0.010	-0.001	0.001	-0.013	-0.017
	Personal care	-0.098	-0.080	-0.107	-0.078	0.008	-0.074	-0.166	-0.177	-0.139	-0.112
	Meals	-0.017	-0.009	0.007	0.008	0.035	-0.037	0.001	0.056	0.035	0.029
	Paid work	0.070	0.237	0.192	0.168	0.243	0.082	0.169	0.165	0.118	0.120
	Study	0.002	-0.086	-0.114	0.004	-0.068	0.085	-0.132	0.089	-0.060	0.062
	Housework	-0.360	-0.609	-0.260	-0.330	-0.418	-0.544	-0.165	-0.320	-0.232	-0.273
	Care for others	-0.528	-0.607	-0.507	-0.645	-0.743	-0.775	-0.527	-0.672	-0.590	-0.481
	Active leisure	0.200	0.090	0.187	0.030	0.319	0.238	-0.039	0.222	0.187	0.470
	Screen-based leisure	0.121	0.058	0.110	0.151	0.115	0.132	0.168	0.136	0.163	0.046
	Other leisure	0.037	0.069	-0.019	-0.008	0.043	0.030	-0.047	-0.079	0.019	0.090
30–44	Sleeping	-0.004	-0.007	-0.014	-0.028	-0.012	0.006	-0.015	-0.008	-0.018	-0.019
	Personal care	-0.019	0.003	-0.095	-0.038	0.047	0.014	-0.133	-0.123	-0.140	-0.123
	Meals	-0.007	-0.009	0.004	-0.022	0.020	-0.031	-0.016	-0.011	0.022	0.031
	Paid work	0.237	0.331	0.205	0.342	0.244	0.328	0.189	0.279	0.191	0.204
	Study	-0.134	-0.181	-0.235	-0.037	-0.012	-0.036	-0.155	0.089	-0.049	-0.402
	Housework	-0.411	-0.646	-0.280	-0.338	-0.448	-0.733	-0.245	-0.341	-0.229	-0.305
	Care for others	-0.293	-0.435	-0.368	-0.314	-0.397	-0.617	-0.345	-0.369	-0.274	-0.284
	Active leisure	0.180	0.139	0.146	0.086	0.239	0.117	0.071	0.076	0.122	0.285
	Screen-based leisure	0.145	0.158	0.153	0.103	0.137	0.077	0.180	0.120	0.130	0.107
	Other leisure	0.039	0.074	-0.063	-0.066	0.022	0.049	-0.028	-0.095	-0.048	-0.011
45–64	Sleeping	0.006	-0.003	-0.012	-0.016	-0.002	0.001	-0.002	-0.017	-0.016	-0.012
	Personal care	-0.022	0.013	-0.086	-0.068	0.007	-0.005	-0.081	-0.117	-0.157	-0.162
	Meals	-0.002	0.000	0.020	-0.026	0.039	0.000	0.007	0.008	0.043	0.055
	Paid work	0.298	0.359	0.161	0.355	0.201	0.279	0.064	0.180	0.151	0.137
	Study	-0.078	-0.451	-0.296	0.169	-0.103	-0.134	-0.331	-0.133	0.059	-0.266
	Housework	-0.504	-0.600	-0.245	-0.333	-0.366	-0.696	-0.163	-0.266	-0.176	-0.256
	Care for others	-0.232	-0.269	-0.109	-0.343	-0.362	-0.555	-0.004	-0.192	-0.245	-0.215
	Active leisure	0.201	0.309	0.205	0.057	0.290	0.101	-0.031	0.077	0.076	0.279
	Screen-based leisure	0.095	0.140	0.093	0.104	0.099	0.043	0.166	0.140	0.111	0.080
	Other leisure	0.071	0.081	-0.020	-0.050	0.050	0.028	-0.045	-0.082	-0.088	-0.079
65+	Sleeping	0.012	-0.005	-0.006	-0.009	0.004	-0.009	0.002	-0.004	-0.008	0.002
	Personal care	-0.033	0.039	-0.035	-0.025	0.080	0.031	-0.065	-0.055	-0.189	-0.154
	Meals	0.025	0.034	0.024	0.057	0.054	0.037	0.012	0.026	0.038	0.026
	Paid work	0.466	0.684	0.435	0.865	0.283	0.315	0.678	0.560	0.447	0.224
	Study	-0.135	0.475	-0.342	0.266	0.142	-0.132	0.066	-0.119	0.249	0.598
	Housework	-0.433	-0.462	-0.223	-0.194	-0.315	-0.514	-0.163	-0.174	-0.110	-0.244
	Care for others	-0.004	-0.006	0.052	-0.164	-0.224	-0.232	-0.446	-0.020	-0.074	-0.026
	Active leisure	0.371	0.478	0.333	0.303	0.279	0.240	0.179	0.278	0.164	0.319
	Screen-based leisure	0.034	0.114	0.053	0.130	0.080	0.085	0.081	0.091	0.096	0.092
	Other leisure	0.121	0.083	0.022	-0.035	0.047	-0.003	-0.032	-0.057	-0.069	-0.057

Source: Own calculations from the Multinational Time Use Study [32].

In the case of *paid work*, relative time-use gender gaps differed across ages and countries (see Fig 3). Gender gaps were largest and more different cross-nationally within the extreme age groups (10–17 and 65 and older). Yet, these results need to be taken with caution, as very few people engaged in paid work at these opposite groups of age. Gender gaps in paid work time were flatter in the age groups between 18 and 64, except for Italy and the Netherlands where the indicator took higher values, with men engaging disproportionately in paid work (around +0.35).

For *housework*, changes across age groups were most evident in South Korea, Southern Europe (especially Italy) and Hungary (see Fig 3). In all groups, women did more housework, regardless of age. During early and middle adulthood (i.e. ages 30–44) the indicator took values that got closer to -1, that is women doing more housework, particularly in Italy and South Korea. Finland and Canada showed lower relative gender gaps in housework time. As for *caring work*, the higher gender gaps corresponded to South Korea across most age groups, with gaps ranging between -0.75 and -0.25 among adults. Gaps for the 10–17 age group seemed sensitive to low participation rates, meaning that results for the youngest group need to be taken with caution.

Finally, for *personal care*, women did more of it across countries and ages (negative values), with cross-country differences increasing at the age of 65 or older, showing gaps in favor of women in Canada and the US (around -0.1), but interestingly in favor of men in Hungary (+0.08) (see Fig 3). Differences in *study* time were largest for the older groups, even if study time was rare for the elderly. Interestingly, for the 30–44 age group, the US presented the largest gender gaps in study time (women did more of it) and for the 65 or older group (but here men did more of it). Gender gaps in *sleeping* and *meals* remained around 0 in all ten countries.

#### Regression analyses

**Fig 4:**
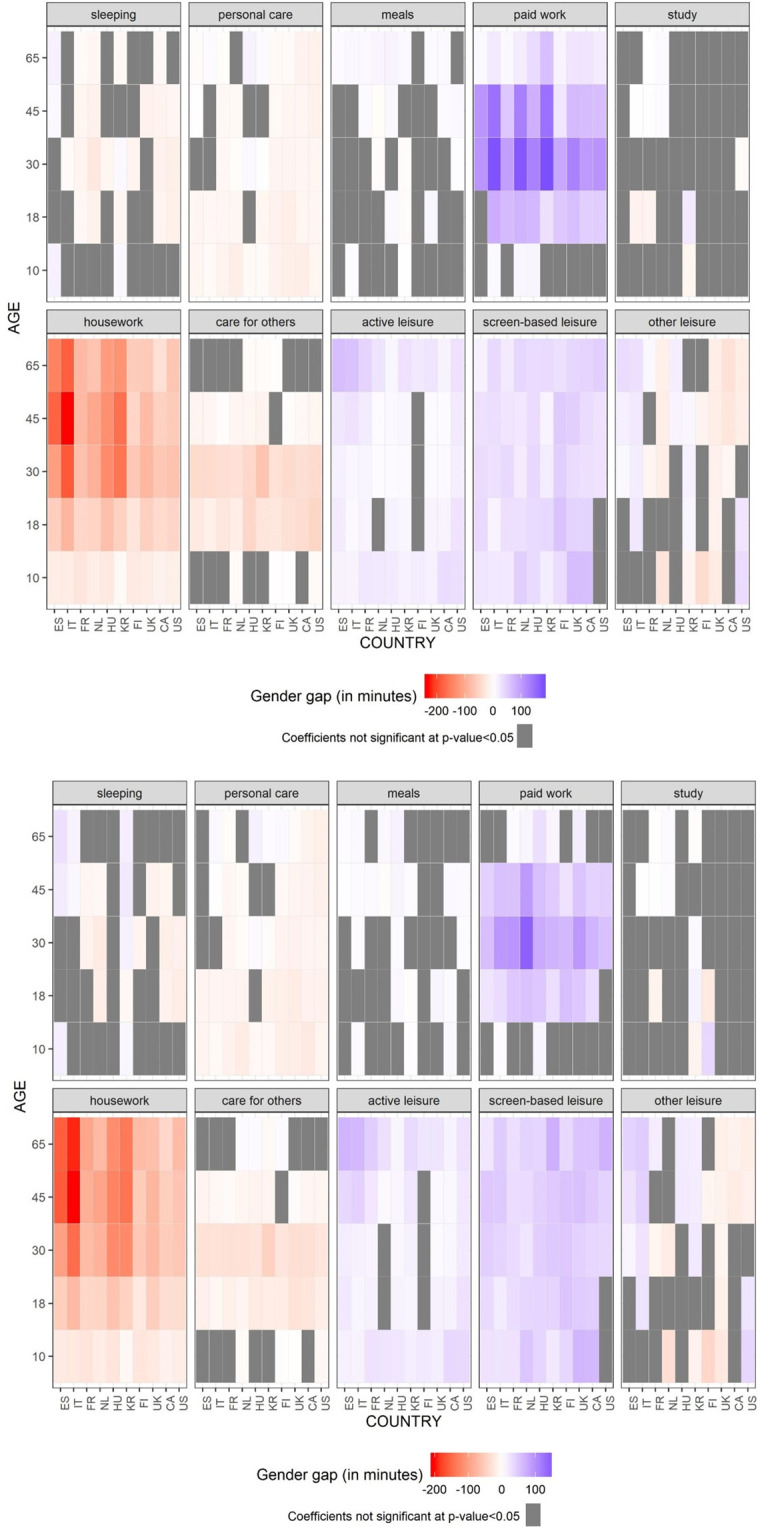
Gender differences in time use. Panel A. Without adjusting for socioeconomic and demographic factors. Panel B. Adjusting for socioeconomic and demographic factors. Source: Own calculations from the Multinational Time Use Study [32]. Notes: Estimates for Panel A are available in Table A3 in S1 Appendix and for Panel b in Table A4 in S1 Appendix. Estimates correspond to the coefficient for Category Men (ref = Women) from the OLS regressions by country and group of age. Panel A also control by day of the week. Panel B also control by day of the week, educational attainment, employment status, children in the household and union status.

Fig 4 presents the results of the regression analyses. In Panel A, we present the statistical association between gender and time use for our ten activities of study and with separated models for each group age and country, without adding demographic and socioeconomic controls, and only controlling for day of the week. In Panel B, we present the same analyses, adding also a control of day of the week, but in this case we control for multiple socioeconomic and demographic variables, including education, employment status, union status and number of children at home. Combinations in grey denote the coefficients that are not significant at 95% level of significance. All coefficients and levels of statistical significance are presented in Tables A3 and A4 in S1 Appendix.

Fig 4 shows clear differences in the intersection of age, gender and country in predicting variations in time use. Panel A shows a very similar picture to the one presented in Fig 2, showing clear gendered patterns across age groups in housework, work and leisure, where the gender coefficients are statistically significant (p < 0.05) in most countries and age groups, but with some exceptions where gender differences are not statistically significant, including the ages 10–17 for paid work (Canada, Finland, South Korea, Spain, UK, US) and for leisure the Netherlands (ages 45–64 and 65 and older) and France (ages 10–17). For housework, the gender gap is statistically significant (p < 0.05) in all countries and age groups, but the gaps are stronger in Italy, South Korea and Spain for the age groups 30–44, 45–64 and 65 and older, with the most gender unequal division of housework being observed among Italians for the age group 45–64. Higher gender gaps in paid work are in population in the group of age 30–44, whereas Netherlands and South Korea present the higher coefficients. For screen-based leisure, we observe the highest gender gaps in the extreme age groups (10–17 and 65 and over), while the US was the country with larger gender gaps. For active leisure, the highest gender gaps were found in the group 65 and older, while Spain and Italy were the countries where these gaps were largest.

Panel B of Fig 4 shows that, when socioeconomic and demographic controls are added, the picture of gender differences in time use by age groups shows only some minor changes. The inclusion of controls increases the magnitude of the gender gap in housework, particularly for the population aged 45 and older. On the contrary, gender gaps in paid work generally decrease after adjusting for demographic factors, especially for the age group 30–44. The 30–44 age group includes a high proportion of population living in union with children, and so these demographic controls globally attenuate the relationship between gender and time spent in employment. Regarding leisure, the inclusion of controls leads to a certain increase in the strength of gender gaps in all ages for both active and screen-based leisure. However, we do not observe important changes in the level of significance for these coefficients. Largest changes are observed in screen-based leisure for the older group, with an increase of gender-gaps in all countries. In the case of active leisure, gender gaps in time use increased for the group 45–64 in all countries.

Overall, despite some minor differences between Panel A and Panel B in Fig 4, adding control variables does not generally alter the relation between gender and age in individuals’ time use across countries.

#### Additional analyses

We conducted additional analyses that control for retirement in the older groups of study. We did this by differentiating in the analyses between unemployed, employed, and retired people for our two older groups: ‘45 to 64 years old’ and ‘65 years old or older’. First, Fig A1 in S1 Appendix adds the category retirement compared to unemployed within our multivariate statistical framework, controlling for all other variables of study, including being employed too. These analyses exclude Finland due to having no data on retirement status for this country. Results for the other nine countries reveal that, when differentiating between pensioners, employed and unemployed as controls, observed gender differences in time use remain unchanged.

Second, we conducted additional analyses to compare differences in time use between retired people and unemployed respondent, also adding a coefficient of being employed, controlling for various demographic factors, including age, gender, and country of study, again excluding Finland from the models. In Table A6 in S1 Appendix we observe some net differences between unemployed and retired people. For the age group of 45 to 64 years old we find that, compared to the retired, the unemployed spent 2 minutes less eating, 12 minutes more in housework, 21 minutes less on physical care, 16 minutes less on non-screen leisure, 1 minute less studying, and 26 minutes more in paid work (*p* < 0.05). Differences between retired and unemployed individuals for this age group were not statistically significant for care work, screen-based leisure, personal care, and sleeping. The observed differences between the unemployed and retired in paid work capture that retired people in the 45–64 age groups may have access to a pension. By contrast, the unemployed may eventually have to engage in casual paid work activities to make ends meet (typically through informal activities), particularly when unemployment benefits are limited or unavailable. However, we must stress that additional analyses (not shown) reveal that the proportion of unemployed people who spent some time in paid work in our sample is very low.

For the group of 65 years or older, differences between become statistically insignificant for meals, while we observe that the unemployed slept 8 daily minutes more than retired respondents (*p* < 0.05), net of all demographic controls. We interpret these results as a sign that retirement is associated with different life conditions compared to being a non-retired non-working person (e.g., unemployed people may have to engage more frequently in informal work activities than pensioners to make ends meet) or due to selection (e.g., unemployed people may have a more disadvantaged socioeconomic background than pensioners). Understanding these specific mechanisms goes beyond the scope of the present study.

Future studies should further address such interesting differences by carefully analyzing the interplay between gender, age, and country, considering existing variations in retirement and work policies related to gendered time-use patterns. Unfortunately, we cannot specifically focus on the phenomenon of retirement for reasons of focus, space, and incomplete data on retirement for some countries. However, and crucially, additional analyses reveal that, after controlling for retirement in our models, the observed country and gender differences in our main analyses remain the same. Therefore, the main results of this paper remain do not change irrespective of whether we control for being a pensioner or not.

## Discussion

This article provides an exhaustive large-scale study of gender differences in time-use across age groups, using recent high-quality data across ten industrialized countries spanning the regions of Asia, Europe and North America. We make three key contributions to the international cross-national time use and gender literatures by (i) examining detailed time-diary data from childhood to old age, (ii) studying eight activities with key well-being implications, and (iii) showing age-specific gender gaps at both absolute (minute gap) and relative levels (percentage gaps). Our results clearly indicate that gender differences in time use differ by activities and across age groups. Yet, the magnitude of these gender-age interaction differs remarkably across countries with different policy contexts and gender norms.

First, our study shows that gender gaps in time use are small in essential activities such as sleeping and meals, moderate in leisure time, and highest in paid work (with men doing more of it) and domestic work (with women doing more of it). Yet, these gender gaps were largest in South Korea, Hungary and Southern Europe (more in Italy than in Spain). Western Europe (i.e. France, Netherlands) showed intermediate gender gaps in time use. Anglo-Saxon countries (more in Canada than the US and UK) and Finland showed the lowest gender differences in time-use patterns. These cross-country findings add new relevant evidence on the most robust and recent available time-diary cross-country data on gendered time use patterns. Lower differences in more essential activities reveal that gender gaps increase in activities that are more gendered stereotypical (especially paid work and domestic work) and in activities that depend more on the remaining time after basic and mandatory activities are considered (leisure time).

Second, we found important cross-country gender variations in time use across age groups that represent key variations in lifestyles and life-course stages. Gender gaps in housework and care work were largest in the adult population over 29 and 44 years old, narrowing substantially among the elderly (from 65 years old onward). Italy, and especially South Korea, exhibited the largest gender gaps in domestic work, while Canada and Finland presented smaller gaps for these age groups in these activities. Furthermore, we found gender gaps in paid work to be largest during early/middle and middle/late adulthood (aged 30–44 and 45–64), and this was especially true for the Netherlands. Study time showed mixed patterns across countries and gender. Interestingly, both boys and girls in South Korea (ages 10–17) spent much more time studying than their counterparts in other countries, at the costs of their leisure time, showing an interesting finding to be explored in future literature.

Third, we conducted various statistical analyses to establish if findings are sensitive to demographic and socioeconomic factors. Our results generally remain stable when sociodemographic factors are considered, although factors such as the presence of children or being in a union moderate the association between gender and those activities where this association is stronger. Early/middle adulthood (ages 30–44) and middle/late adulthood (ages 45–64) are the ages where the gender gap in time use is most affected by the consideration of demographic and socioeconomic variables in the analyses, but the direction of this variation differs remarkably between activities and countries. Gender gaps in employment time decrease when controls are added, whereas they are larger in the case of housework. This fact indicates that gaps in housework work and paid work are strongly driven by family characteristics (e.g., parenthood transitions) [3], although we still observe persistent gender gaps in both domestic work and paid work across countries when such family characteristics are considered. By contrast, gender gaps in leisure activities increase after adjusting for sociodemographic factors, indicating that mechanisms of gender inequalities in leisure follow different patterns than those linked to paid work, care work and housework. This pattern is observed in all countries, although it is clearer in countries with overall higher gender inequalities, like Hungary, Italy, South Korea and Spain.

The findings of our study suggest that policy and cultural contexts can critically shape the opportunities that men and women encounter to engage in different activities with key well-being and health implications. We found some relevant cross-national differences in time use in activities such as sleeping and personal care, which are known to be associated with physical health and mental well-being [22–28], even if age-gender interactions for these activities did not differ cross-nationally. Yet, differences by country, age, and gender were significant when examining active leisure (a healthy activity to which women devote less time) and screen-based leisure (a potentially risky activity to which men devote more time) [25–28]. These findings contribute to international debates on health and well-being.

Interestingly, personal care does not differ significantly by age in our analyses. It is important to clarify that all activities like washing up, dress up, care for oneself, but also doctor visits, are included as personal care. By contrast, being in the hospital to care for others, and related activities, would be care work. We cannot differentiate between time travelling to hospital and other traveling with our data. Thus, all types of traveling are included in the traveling category (see the methods section). We hope future studies will be able to further investigate these important questions with more detail.

In general, we show that the national context plays an important role in moderating existing time-use gender gaps across the life course. We show that women in South Korean (an East Asian case), Italy (a Southern European case), followed by Hungary (an East-Central European case) and Spain (a Southern European case) are countries where women have higher constraints to spend time in non-domestic activities. Also, these gender gaps across countries are particularly strong in the ages associated with higher caring responsibilities (ages 30–44), with interesting patterns observed in specific countries. For example, Dutch women suffer the strongest paid work penalties of all countries from early adulthood (18–29 years old) to early/middle adulthood (30–44), capturing the institutionalization of female part-time employment in the country [40]. Contrary to less gender egalitarian countries in terms of gender politics and gender ideologies, Anglo-Saxon countries (and particularly Canada in our study) and Nordic European countries (i.e., Finland) reveal higher gender equality in time use than other countries, but especially regarding the most sensitive age groups with respect to gender inequalities (early/middle and middle/late adulthood). These results are important to situate public policy debates on the importance of targeting gender gaps in time use during early/middle adulthood, when risks of gender inequalities are highest, and particularly so in countries providing poorer support and resources to women in these age groups.

Our study offers a new understanding of how gender and age intersect which contribute to recent cross-national studies on gender gaps in time use. Our results help to contextualize existing scholarship that indicates cross-country variations in life satisfaction and well-being by highlighting the importance of age in shaping these gender disparities in how men and women use their time every day [3]. Also, by bringing together a time-use and life-course approach, our study provides a picture that contributes to cross-national research that has often focused on very specific age groups separately, including childhood and adolescence [18], early adulthood [41] or late adulthood [42], while complementing exceptional older studies that adopted a similar approach to our study [10].

We should acknowledge four types of limitations in our study, despite our contribution to the gender, time use and cross-national literatures. First, this study only uses the age of individuals to follow the life course. Not all individuals are in the same conditions at a specific age and future research should add other transition factors (i.e., union status, parenthood, retirement) to capture better the differences in time use during the life course and explore the causes of gender inequalities across national contexts. Our study conducts multiple regression analyses controlling for several demographic variables on such life-course stages, with results remaining quite consistent after these adjustments are added. Age is an interesting variable because it is constant across the world, despite people aging differently across different regions of the globe. The fact that we find differences between countries when comparing the same age groups, but also some consistent patterns, does reflect the interest of examining age variations to understand gender differences in time use. Future studies should focus on other indicators related to the life course (not only age) and more closely inspect issues related to retirement and youth unemployment, which vary significantly across countries.

Second, this study is limited to cross-sectional data collected around 2010 and individuals in each group of age are not the same. The availability of longitudinal data would provide information of the same individuals at different moments of their life course and would improve the analysis of changes on time use throughout the life course. However, at this stage, high-quality longitudinal time-use data with rich measures from diary data are very scarce and limited to very few countries, which does not allow to conduct a large comparable cross-national analysis across various countries and regions.

Third, future related research should consider a 24-hour analytical framework by applying some alternative techniques that can more explicitly integrate all activities conducted over the day (Bauman et al. 2019). Also, we must remember that activities are not fully comparable in terms of costs and benefits, which needs to be considered regarding the units of time. For example, the benefits of spending one hour exercising are not comparable to the benefits of spending one hour sleeping, as the latter is a much longer activity than the former. We hope future studies will ask similar questions than the ones we are asking in this study by adopting complementary methods that account for the nature of time-use tradeoffs that men and women face within a 24-hour context.

Fourth, this is an observational study that is subject to issues of selection and which does not have complete relevant information on demographic and socioeconomic variables. Our study presents multivariate analyses that adjust for employment status. This allows us to analytically disentangle the degree to which men and women differ in their time use after accounting for selection in employment. Yet, there may be other issues of selection that we cannot account for in our data (e.g. type of contract, specific occupation, employment trajectory) and that may still explain differences. This limits our ability to explain why gender differences in time use exist across age groups across the ten countries included in this study. Future studies should pay more attention to these issues by using surveys that, despite adding less countries and potentially poorer quality data on time use (e.g., some longitudinal surveys), do contain better data on employment trajectories and repeated observations to account for unobserved heterogeneity.

To conclude, and despite having some shortcomings that we acknowledge above, our study opens an important window to future studies and approaches on gender differences in time use. Future research should combine our approach with one that accounts for different periods to analyze the interaction between gender, age and time use, not only across countries, but also over recent decades by considering cohort and period effects [18, 43]. For example, recent studies conducted during the COVID-19 pandemic lockdowns reveal persistent time use differences between women and men due to the new responsibilities derived from the COVID-19 emergency and temporary closure of schools [44, 45]. Future studies will be able to cover these more recent years in relation to the age and gender interactions in time use allocation across countries. We hope scholars will further collect rich time-use data from new countries across policy and cultural contexts, especially from low- and middle-income countries. This way scholars will be able to offer new understandings of how national contexts shape gender differences in time use across age groups and to assert in which direction gender gaps in time use are moving to.

## Supporting information

S1 Appendix (DOCX)
