# Correction: Gender differences in time use across age groups: A study of ten industrialized countries, 2005–2015

**DOI:** 10.1371/journal.pone.0307860

**Published:** 2024-07-23

**Authors:** Joan García Román, Pablo Gracia

The numbers in Table 1 are incorrect. Please see the correct Table 1 here.

**Table 1:** Average time and standard deviation by activity. Men’s and Women’s daily minutes by country.

**SEX**	**ACTIVITY**	**Spain**	**Italy**	**France**	**Netherlands**	**Hungary**	**South Korea**	**Finland**	**UK**	**Canada**	**US**
Men	Sleeping	524	518	501	508	513	474	517	512	496	510
		(124,9)	(113,1)	(107,7)	(104,8)	(118)	(96,7)	(117,8)	(129)	(121,8)	(134,2)
	Personal care	49	53	49	53	79	71	43	51	33	43
		(32)	(34,5)	(38,3)	(45,3)	(52,2)	(31,4)	(36,5)	(40,1)	(37,8)	(57,7)
	Meals	100	106	136	76	103	69	79	89	62	58
		(47,6)	(46,3)	(73,9)	(48,9)	(52,8)	(46,9)	(44,3)	(64,9)	(57)	(50,3)
	Paid work	179	210	181	211	207	253	166	181	227	234
		(254,8)	(259,3)	(244,3)	(261,9)	(247,5)	(248,3)	(245,4)	(248,3)	(268,2)	(269,4)
	Study	39	37	13	61	31	90	35	33	29	21
		(122)	(117,9)	(74,4)	(140,9)	(107,1)	(197,1)	(111,5)	(108,8)	(107,5)	(92,3)
	Housework	78	50	118	88	95	31	110	99	115	86
		(99,2)	(91,3)	(124,7)	(106,5)	(105,7)	(55,1)	(116,6)	(107,6)	(138,6)	(117,5)
	Care for others	27	16	19	16	17	10	11	13	23	30
		(72,2)	(51,3)	(57,8)	(50,3)	(49,4)	(35,3)	(41,9)	(43,1)	(73,3)	(76,8)
	Active leisure	69	57	57	37	21	36	51	41	39	47
		(96,5)	(93)	(95,2)	(73)	(58,7)	(64,8)	(81,1)	(72,9)	(83,6)	(96,1)
	Screen-based leisure	173	140	163	128	189	151	190	199	172	188
		(140,1)	(109,4)	(137,4)	(119,9)	(136,1)	(132,8)	(148,5)	(164,3)	(167,1)	(180,5)
	Other leisure	127	160	123	184	120	160	158	121	165	140
		(133,6)	(136,1)	(132,1)	(162,7)	(121)	(104,5)	(147,8)	(134,8)	(180,6)	(162,9)
	Travel	72	90	79	79	65	95	68	77	79	77
		(66)	(67,9)	(83)	(78,9)	(68,5)	(69,2)	(82,1)	(80)	(83,6)	(79,9)
	Other	4	2	1	0	0	2	11	25	3	8
		(32)	(21,2)	(7,6)	(0,5)	(1,1)	(16,7)	(58,1)	(99,7)	(28,5)	(39,8)
Women	Sleeping	521	525	511	522	519	474	519	520	510	524
		(114,4)	(108,1)	(107,1)	(102,9)	(100,8)	(94,3)	(106,4)	(123,7)	(120,6)	(132)
	Personal care	53	54	57	61	75	72	54	64	46	57
		(34,6)	(34,7)	(39,3)	(45)	(52,7)	(38,2)	(36,8)	(45)	(40,1)	(64,7)
	Meals	100	106	132	76	97	71	79	86	58	54
		(45,9)	(43,8)	(70,6)	(49,5)	(46,1)	(41,9)	(43,3)	(61,3)	(55)	(47,5)
	Paid work	106	95	122	116	126	143	130	116	161	165
		(193,4)	(188)	(203,6)	(198,1)	(197,1)	(208,5)	(209,7)	(203,1)	(231,7)	(235,7)
	Study	37	39	16	58	28	81	32	32	29	20
		(119,9)	(121,2)	(81,7)	(136,6)	(102,3)	(187,4)	(101,7)	(104,7)	(107,7)	(89,5)
	Housework	207	226	195	165	218	155	163	169	169	152
		(151,5)	(172,6)	(134,7)	(135)	(139,8)	(124,7)	(123,6)	(130)	(148,7)	(142,2)
	Care for others	47	33	33	34	39	42	24	30	44	54
		(95,5)	(77,3)	(71,9)	(74,7)	(89,3)	(87,4)	(73,3)	(76,7)	(102,6)	(100,3)
	Active leisure	44	33	35	27	11	26	45	29	29	25
		(68,3)	(60,8)	(64,6)	(59,1)	(38,4)	(51,4)	(66,4)	(56,2)	(66,7)	(57,7)
	Screen-based leisure	147	113	136	98	157	132	143	154	136	163
		(121,4)	(96,7)	(122,6)	(95,9)	(115,1)	(111,8)	(116,9)	(130,1)	(140,7)	(162,7)
	Other leisure	113	143	128	203	116	156	174	140	186	149
		(122,6)	(122,9)	(125)	(153,3)	(113,9)	(107,4)	(139)	(132,7)	(179,9)	(159,5)
	Travel	64	73	73	79	55	87	68	72	71	73
		(61,4)	(64)	(77,7)	(78,4)	(60,2)	(67,9)	(80,2)	(73,7)	(77,1)	(78,7)
	Other	1	0	1	0	0	0	9	28	2	6
		(16,2)	(6,6)	(7,7)	(0,4)	(1,1)	(7)	(41,2)	(103,4)	(22,5)	(28,8)

Source: Own calculations from the Multinational Time Use Study [32]
