# Supplementary material for: Gender differences in time use across age groups: A study of ten industrialized countries, 2005–2015
Source: PLoS One. 2022 Mar 9;17(3):e0264411. doi: 10.1371/journal.pone.0264411 (PMC8906609; doi:10.1371/journal.pone.0264411)
Supplement: S1 Appendix — (DOCX) [file pone.0264411.s001.docx]

**ANNEX**

Table A1. Sample size by country, gender and group of age

| **SEX** | **AGE** | **Spain** | **Italy** | **France** | **Netherlands** | **Hungary** | **South Korea** | **Finland** | **UK** | **Canada** | **US** |
| --- | --- | --- | --- | --- | --- | --- | --- | --- | --- | --- | --- |
| Men | 10-17 | 747 | 1703 | 357 | 1071 | 266 | 3326 | 454 | 864 | 256 | 299 |
|  | 18-29 | 1101 | 2520 | 1375 | 1036 | 619 | 2738 | 442 | 1054 | 762 | 796 |
|  | 30-44 | 2322 | 4568 | 3327 | 1897 | 932 | 5330 | 685 | 1553 | 1540 | 1728 |
|  | 45-64 | 3072 | 5574 | 4872 | 2149 | 1310 | 5778 | 1329 | 2393 | 2643 | 2081 |
|  | 65+ | 1734 | 3850 | 2927 | 938 | 637 | 2256 | 594 | 1553 | 1500 | 908 |
|  | Total | 8976 | 18215 | 12858 | 7091 | 3764 | 19428 | 3504 | 7417 | 6701 | 5812 |
|  |  |  |  |  |  |  |  |  |  |  |  |
| Women | 10-17 | 689 | 1587 | 405 | 1092 | 262 | 3088 | 448 | 948 | 232 | 282 |
|  | 18-29 | 1194 | 2499 | 1694 | 1512 | 611 | 3026 | 518 | 1349 | 979 | 1014 |
|  | 30-44 | 2746 | 4841 | 3763 | 2086 | 994 | 5922 | 825 | 1946 | 1985 | 2188 |
|  | 45-64 | 3388 | 5930 | 5587 | 2730 | 1733 | 5926 | 1443 | 2634 | 3354 | 2457 |
|  | 65+ | 2302 | 5162 | 3590 | 917 | 1026 | 3136 | 717 | 1803 | 2139 | 1507 |
|  | Total | 10319 | 20019 | 15039 | 8337 | 4626 | 21098 | 3951 | 8680 | 8689 | 7448 |

Source: Multinational Time Use Study [32].

Table A2. Respondent percentage spending at least 1 daily minute in activities by country, gender and age

|  |  | **Spain** | | **Italy** | | **France** | | **Netherlands** | | **Hungary** | | **South Korea** | | **Finland** | | **UK** | | **Canada** | | **US** | |
| --- | --- | --- | --- | --- | --- | --- | --- | --- | --- | --- | --- | --- | --- | --- | --- | --- | --- | --- | --- | --- | --- |
| AGE | ACTIVITY | M | W | M | W | M | W | M | W | M | W | M | W | M | W | M | W | M | W | M | W |
| 10-17 | Sleeping | 100 | 100 | 100 | 100 | 100 | 100 | 100 | 100 | 100 | 100 | 100 | 100 | 100 | 100 | 100 | 100 | 100 | 100 | 100 | 100 |
|  | Personal care | 97 | 98 | 98 | 99 | 97 | 97 | 92 | 97 | 100 | 100 | 100 | 100 | 90 | 94 | 94 | 97 | 90 | 93 | 87 | 91 |
|  | Meals | 100 | 100 | 100 | 100 | 98 | 99 | 94 | 95 | 100 | 100 | 99 | 98 | 97 | 98 | 97 | 97 | 84 | 76 | 91 | 93 |
|  | Work | 0 | 1 | 2 | 1 | 7 | 6 | 14 | 10 | 16 | 8 | 1 | 1 | 5 | 6 | 6 | 5 | 17 | 22 | 12 | 11 |
|  | Study | 71 | 72 | 73 | 77 | 61 | 62 | 76 | 79 | 63 | 61 | 95 | 96 | 59 | 56 | 57 | 59 | 59 | 65 | 59 | 67 |
|  | Housework | 49 | 66 | 41 | 59 | 42 | 68 | 38 | 56 | 64 | 70 | 27 | 38 | 53 | 76 | 63 | 77 | 43 | 60 | 59 | 64 |
|  | Care for others | 4 | 6 | 2 | 4 | 6 | 12 | 3 | 9 | 10 | 10 | 4 | 6 | 2 | 6 | 1 | 4 | 2 | 5 | 21 | 27 |
|  | Active leisure | 50 | 41 | 46 | 39 | 41 | 30 | 46 | 37 | 44 | 30 | 35 | 12 | 44 | 46 | 40 | 31 | 42 | 24 | 49 | 37 |
|  | Screen-based leisure | 92 | 92 | 92 | 89 | 85 | 84 | 82 | 73 | 91 | 94 | 83 | 79 | 90 | 94 | 93 | 86 | 92 | 80 | 85 | 79 |
|  | Other leisure | 78 | 80 | 85 | 90 | 68 | 76 | 83 | 92 | 76 | 79 | 99 | 99 | 81 | 96 | 79 | 86 | 82 | 83 | 88 | 81 |
|  | Travel | 86 | 87 | 95 | 96 | 85 | 82 | 88 | 87 | 86 | 81 | 97 | 96 | 80 | 84 | 82 | 84 | 92 | 92 | 94 | 87 |
| 18-29 | Sleeping | 100 | 100 | 100 | 100 | 100 | 100 | 100 | 100 | 100 | 100 | 100 | 100 | 100 | 100 | 100 | 100 | 100 | 100 | 100 | 100 |
|  | Personal care | 96 | 98 | 97 | 99 | 94 | 95 | 96 | 97 | 100 | 100 | 100 | 99 | 93 | 98 | 91 | 96 | 84 | 84 | 83 | 86 |
|  | Meals | 100 | 100 | 99 | 100 | 99 | 99 | 94 | 92 | 100 | 100 | 93 | 95 | 97 | 97 | 95 | 94 | 83 | 80 | 89 | 87 |
|  | Work | 33 | 33 | 44 | 32 | 51 | 38 | 53 | 43 | 43 | 29 | 47 | 43 | 41 | 36 | 48 | 38 | 47 | 41 | 51 | 43 |
|  | Study | 27 | 28 | 25 | 31 | 16 | 21 | 27 | 30 | 27 | 31 | 38 | 36 | 19 | 26 | 18 | 17 | 19 | 23 | 15 | 17 |
|  | Housework | 55 | 82 | 40 | 80 | 70 | 86 | 59 | 79 | 70 | 89 | 45 | 75 | 80 | 88 | 77 | 90 | 74 | 85 | 73 | 87 |
|  | Care for others | 10 | 22 | 6 | 17 | 15 | 31 | 7 | 18 | 9 | 24 | 7 | 21 | 12 | 22 | 12 | 29 | 13 | 28 | 31 | 57 |
|  | Active leisure | 46 | 40 | 38 | 38 | 28 | 25 | 20 | 22 | 23 | 19 | 28 | 21 | 41 | 51 | 34 | 25 | 29 | 26 | 36 | 21 |
|  | Screen-based leisure | 88 | 87 | 80 | 79 | 82 | 80 | 72 | 73 | 88 | 85 | 88 | 86 | 88 | 88 | 84 | 83 | 79 | 74 | 79 | 80 |
|  | Other leisure | 80 | 79 | 89 | 87 | 72 | 77 | 84 | 89 | 81 | 78 | 99 | 98 | 78 | 87 | 71 | 78 | 80 | 81 | 78 | 75 |
|  | Travel | 88 | 87 | 96 | 93 | 90 | 89 | 87 | 89 | 88 | 82 | 95 | 93 | 82 | 88 | 85 | 86 | 92 | 91 | 94 | 89 |
| 30-44 | Sleeping | 100 | 100 | 100 | 100 | 100 | 100 | 100 | 100 | 100 | 100 | 100 | 100 | 100 | 100 | 100 | 100 | 100 | 100 | 100 | 100 |
|  | Personal care | 97 | 97 | 98 | 98 | 92 | 95 | 97 | 98 | 100 | 100 | 100 | 99 | 94 | 97 | 95 | 97 | 83 | 86 | 83 | 86 |
|  | Meals | 99 | 100 | 99 | 100 | 99 | 99 | 94 | 93 | 100 | 100 | 95 | 97 | 97 | 99 | 96 | 95 | 85 | 84 | 91 | 92 |
|  | Work | 56 | 43 | 72 | 45 | 65 | 52 | 67 | 43 | 71 | 52 | 79 | 47 | 60 | 46 | 63 | 43 | 64 | 49 | 66 | 50 |
|  | Study | 5 | 7 | 1 | 2 | 2 | 3 | 10 | 10 | 3 | 4 | 7 | 8 | 2 | 5 | 3 | 4 | 5 | 6 | 2 | 6 |
|  | Housework | 73 | 95 | 60 | 95 | 76 | 95 | 81 | 96 | 80 | 98 | 48 | 97 | 90 | 98 | 86 | 96 | 80 | 94 | 78 | 93 |
|  | Care for others | 44 | 62 | 33 | 57 | 41 | 63 | 45 | 63 | 33 | 55 | 33 | 74 | 40 | 58 | 41 | 61 | 44 | 59 | 52 | 72 |
|  | Active leisure | 41 | 36 | 32 | 28 | 32 | 29 | 23 | 23 | 15 | 9 | 30 | 26 | 43 | 45 | 33 | 33 | 27 | 29 | 34 | 31 |
|  | Screen-based leisure | 87 | 84 | 81 | 75 | 82 | 76 | 77 | 76 | 89 | 86 | 87 | 87 | 89 | 85 | 87 | 86 | 79 | 74 | 81 | 78 |
|  | Other leisure | 76 | 77 | 83 | 80 | 69 | 75 | 86 | 93 | 72 | 70 | 99 | 98 | 87 | 94 | 71 | 81 | 73 | 79 | 72 | 76 |
|  | Travel | 91 | 90 | 96 | 93 | 88 | 87 | 81 | 86 | 90 | 82 | 96 | 93 | 83 | 85 | 89 | 87 | 94 | 91 | 94 | 91 |
| 45-64 | Sleeping | 100 | 100 | 100 | 100 | 100 | 100 | 100 | 100 | 100 | 100 | 100 | 100 | 100 | 100 | 100 | 100 | 100 | 100 | 100 | 100 |
|  | Personal care | 97 | 98 | 98 | 97 | 94 | 96 | 97 | 97 | 100 | 100 | 100 | 99 | 93 | 97 | 94 | 97 | 81 | 87 | 79 | 87 |
|  | Meals | 100 | 100 | 100 | 100 | 100 | 100 | 93 | 92 | 100 | 100 | 97 | 97 | 99 | 99 | 97 | 97 | 90 | 87 | 93 | 91 |
|  | Work | 48 | 31 | 56 | 32 | 47 | 39 | 52 | 34 | 64 | 50 | 76 | 49 | 46 | 44 | 51 | 40 | 54 | 43 | 57 | 47 |
|  | Study | 3 | 4 | 1 | 1 | 1 | 2 | 12 | 10 | 1 | 2 | 3 | 4 | 2 | 3 | 2 | 3 | 2 | 3 | 1 | 2 |
|  | Housework | 70 | 97 | 67 | 98 | 83 | 96 | 83 | 97 | 85 | 99 | 51 | 98 | 91 | 98 | 88 | 98 | 83 | 95 | 77 | 91 |
|  | Care for others | 20 | 29 | 17 | 28 | 16 | 23 | 14 | 23 | 12 | 22 | 12 | 35 | 10 | 12 | 12 | 19 | 12 | 17 | 26 | 36 |
|  | Active leisure | 53 | 47 | 42 | 33 | 43 | 40 | 32 | 31 | 15 | 10 | 45 | 40 | 48 | 57 | 39 | 40 | 32 | 35 | 40 | 38 |
|  | Screen-based leisure | 91 | 87 | 88 | 83 | 84 | 81 | 83 | 78 | 93 | 91 | 91 | 90 | 91 | 89 | 90 | 90 | 85 | 80 | 84 | 82 |
|  | Other leisure | 80 | 78 | 87 | 84 | 77 | 82 | 93 | 95 | 85 | 80 | 99 | 99 | 90 | 96 | 76 | 86 | 79 | 87 | 77 | 82 |
|  | Travel | 88 | 82 | 94 | 89 | 83 | 81 | 81 | 78 | 80 | 77 | 94 | 92 | 80 | 83 | 83 | 85 | 88 | 87 | 88 | 86 |
| 65+ | Sleeping | 100 | 100 | 100 | 100 | 100 | 100 | 100 | 100 | 100 | 100 | 100 | 100 | 100 | 100 | 100 | 100 | 100 | 100 | 100 | 100 |
|  | Personal care | 98 | 98 | 98 | 97 | 95 | 96 | 94 | 97 | 100 | 100 | 100 | 99 | 93 | 96 | 96 | 97 | 73 | 85 | 72 | 85 |
|  | Meals | 100 | 100 | 100 | 100 | 100 | 100 | 90 | 91 | 100 | 100 | 99 | 99 | 100 | 100 | 98 | 99 | 93 | 92 | 96 | 96 |
|  | Work | 2 | 1 | 6 | 1 | 5 | 3 | 7 | 2 | 42 | 31 | 43 | 28 | 5 | 1 | 12 | 5 | 13 | 5 | 14 | 9 |
|  | Study | 1 | 2 | 1 | 0 | 1 | 2 | 12 | 6 | 1 | 1 | 3 | 4 | 2 | 1 | 1 | 2 | 1 | 1 | 1 | 1 |
|  | Housework | 72 | 92 | 78 | 92 | 89 | 97 | 89 | 98 | 86 | 99 | 71 | 95 | 94 | 98 | 94 | 98 | 86 | 95 | 78 | 94 |
|  | Care for others | 14 | 16 | 13 | 15 | 12 | 13 | 10 | 12 | 9 | 13 | 12 | 19 | 3 | 7 | 7 | 7 | 5 | 8 | 15 | 16 |
|  | Active leisure | 73 | 51 | 61 | 34 | 60 | 45 | 43 | 33 | 22 | 16 | 65 | 51 | 61 | 61 | 50 | 39 | 38 | 34 | 46 | 37 |
|  | Screen-based leisure | 94 | 94 | 93 | 89 | 91 | 89 | 89 | 82 | 95 | 95 | 97 | 96 | 92 | 93 | 95 | 93 | 91 | 86 | 89 | 88 |
|  | Other leisure | 85 | 80 | 94 | 91 | 92 | 90 | 95 | 98 | 90 | 90 | 98 | 99 | 95 | 98 | 90 | 94 | 89 | 93 | 85 | 91 |
|  | Travel | 73 | 61 | 81 | 67 | 69 | 60 | 69 | 66 | 61 | 57 | 81 | 81 | 58 | 52 | 72 | 68 | 78 | 70 | 72 | 69 |

Source: Own calculations from the Multinational Time Use Study [32].

Note: values go from 0% 100%.

Table A3. Coefficient for Category Men (ref=Women) from the OLS regressions by country and age

|  |  | ES | | IT | | FR | | NL | | HU | | KR | | FI | | UK | | CA | | US | |
| --- | --- | --- | --- | --- | --- | --- | --- | --- | --- | --- | --- | --- | --- | --- | --- | --- | --- | --- | --- | --- | --- |
| ACT | AGE | coef. Men | sig | coef. Men | sig | coef. Men | sig | coef. Men | sig | coef. Men | sig | coef. Men | sig | coef. Men | sig | coef. Men | sig | coef. Men | sig | coef. Men | sig |
| sleep | 10 | 13.635 | * | 4.715 |  | -0.051 |  | 6.254 |  | -8.132 |  | 8.158 | *** | 8.019 |  | 0.299 |  | -10.835 |  | -10.128 |  |
|  | 18 | 6.670 |  | -2.817 |  | -9.271 | * | -16.046 | ** | -12.548 |  | -9.738 | *** | -0.802 |  | 1.078 |  | -13.996 | * | -18.482 | * |
|  | 30 | -4.351 |  | -6.064 | * | -13.285 | *** | -27.902 | *** | -12.168 | * | 6.255 | *** | -13.981 | * | -7.228 |  | -17.587 | *** | -19.611 | *** |
|  | 45 | 6.571 | * | -3.157 |  | -11.212 | *** | -16.174 | *** | -2.104 |  | 1.214 |  | -1.447 |  | -16.837 | *** | -15.212 | *** | -12.048 | ** |
|  | 65 | 13.540 | ** | -5.376 |  | -6.563 | * | -10.193 | * | 4.064 |  | -8.677 | ** | 2.326 |  | -4.396 |  | -8.674 | * | 2.617 |  |
| personal care | 10 | -12.343 | *** | -6.596 | *** | -14.593 | *** | -20.307 | *** | -7.806 | * | -7.231 | *** | -17.005 | *** | -19.427 | *** | -21.672 | *** | -12.381 | * |
|  | 18 | -10.332 | *** | -9.300 | *** | -11.101 | *** | -9.646 | *** | 1.201 |  | -11.626 | *** | -15.953 | *** | -20.181 | *** | -11.322 | *** | -10.981 | *** |
|  | 30 | -1.833 |  | 0.536 |  | -8.872 | *** | -4.017 | ** | 7.025 | ** | 1.872 | ** | -13.034 | *** | -13.361 | *** | -10.324 | *** | -11.545 | *** |
|  | 45 | -2.151 | ** | 1.365 |  | -8.838 | *** | -7.704 | *** | 1.087 |  | -0.672 |  | -7.755 | *** | -12.844 | *** | -12.520 | *** | -16.650 | *** |
|  | 65 | -3.634 | ** | 4.406 | *** | -4.289 | *** | -3.141 |  | 12.981 | *** | 4.437 | *** | -7.175 | ** | -7.001 | *** | -14.617 | *** | -16.487 | *** |
| meals | 10 | 0.034 |  | 4.316 | * | 4.178 |  | -1.296 |  | -2.096 |  | 2.242 | ** | 0.385 |  | -0.239 |  | 7.003 |  | -2.851 |  |
|  | 18 | -3.210 |  | -1.743 |  | 1.688 |  | 0.964 |  | 6.423 | * | -4.325 | *** | 0.114 |  | 8.653 | ** | 3.342 |  | 2.818 |  |
|  | 30 | -1.308 |  | -1.516 |  | 1.508 |  | -3.326 | * | 3.938 |  | -4.415 | *** | -2.308 |  | -1.319 |  | 2.381 |  | 3.199 | * |
|  | 45 | -0.314 |  | -0.060 |  | 5.666 | *** | -4.155 | ** | 8.048 | *** | 0.057 |  | 1.130 |  | 1.415 |  | 5.369 | *** | 6.230 | *** |
|  | 65 | 5.595 | *** | 8.299 | *** | 7.121 | *** | 10.708 | *** | 11.556 | *** | 6.283 | *** | 2.249 |  | 5.940 | * | 6.263 | ** | 3.725 |  |
| paid work | 10 | -0.556 |  | 6.282 | ** | 8.747 |  | 8.848 | * | 9.917 | ** | 0.033 |  | -1.826 |  | 3.974 |  | -10.668 |  | -0.350 |  |
|  | 18 | 20.044 |  | 82.176 | *** | 68.920 | *** | 75.037 | *** | 70.728 | *** | 30.708 | *** | 55.156 | ** | 63.911 | *** | 48.330 | *** | 49.891 | *** |
|  | 30 | 106.915 | *** | 173.763 | *** | 96.967 | *** | 169.701 | *** | 127.026 | *** | 176.400 | *** | 87.994 | *** | 127.523 | *** | 101.569 | *** | 107.811 | *** |
|  | 45 | 107.438 | *** | 143.490 | *** | 55.423 | *** | 129.560 | *** | 79.279 | *** | 143.839 | *** | 24.727 | * | 70.035 | *** | 67.863 | *** | 64.538 | *** |
|  | 65 | 6.366 | ** | 21.012 | *** | 7.082 | *** | 17.399 | *** | 35.092 | *** | 64.170 | *** | 12.297 | ** | 26.423 | *** | 26.545 | *** | 19.278 | * |
| study | 10 | -13.690 |  | -15.054 |  | -10.669 |  | -3.721 |  | -8.141 |  | -12.789 | ** | 32.689 |  | -3.703 |  | -22.760 |  | -27.857 |  |
|  | 18 | 0.106 |  | -16.488 | ** | -14.396 | * | 0.665 |  | -12.417 |  | 20.682 | *** | -16.294 |  | 9.612 |  | -8.167 |  | 5.163 |  |
|  | 30 | -2.937 |  | -1.554 |  | -1.633 |  | -1.009 |  | -0.155 |  | -0.901 |  | -2.293 |  | 0.932 |  | -1.320 |  | -6.360 | ** |
|  | 45 | -0.844 |  | -1.052 | * | -0.782 | * | 4.260 | ** | -0.424 |  | -1.256 |  | -2.782 |  | -0.803 |  | 0.497 |  | -2.124 |  |
|  | 65 | -0.400 |  | 0.544 |  | -0.824 | * | 4.433 | ** | 0.294 |  | -1.035 |  | 0.213 |  | -0.334 |  | 0.513 |  | 1.372 |  |
| housework | 10 | -22.230 | *** | -23.245 | *** | -30.244 | *** | -20.773 | *** | -27.093 | *** | -7.873 | *** | -25.373 | *** | -23.275 | *** | -15.483 | * | -19.542 | * |
|  | 18 | -59.408 | *** | -89.090 | *** | -49.592 | *** | -52.296 | *** | -77.275 | *** | -56.700 | *** | -30.792 | *** | -57.780 | *** | -45.590 | *** | -49.762 | *** |
|  | 30 | -117.204 | *** | -188.796 | *** | -75.345 | *** | -93.435 | *** | -138.117 | *** | -161.463 | *** | -66.955 | *** | -87.946 | *** | -67.720 | *** | -74.113 | *** |
|  | 45 | -179.448 | *** | -233.557 | *** | -84.760 | *** | -105.734 | *** | -133.958 | *** | -167.447 | *** | -50.735 | *** | -82.354 | *** | -56.707 | *** | -63.940 | *** |
|  | 65 | -148.224 | *** | -183.661 | *** | -86.445 | *** | -73.475 | *** | -119.637 | *** | -128.772 | *** | -62.585 | *** | -64.780 | *** | -38.604 | *** | -69.186 | *** |
| care for tohers | 10 | 0.841 |  | -1.112 |  | 3.106 |  | -6.538 | *** | -1.957 |  | -0.631 |  | -2.203 | * | -1.172 | ** | -8.495 |  | -6.952 | * |
|  | 18 | -25.902 | *** | -18.905 | *** | -29.171 | *** | -22.523 | *** | -48.793 | *** | -32.096 | *** | -29.442 | *** | -38.183 | *** | -42.696 | *** | -51.968 | *** |
|  | 30 | -43.868 | *** | -44.250 | *** | -38.686 | *** | -36.517 | *** | -48.292 | *** | -71.338 | *** | -36.838 | *** | -43.992 | *** | -40.079 | *** | -43.703 | *** |
|  | 45 | -13.229 | *** | -10.508 | *** | -3.898 | ** | -11.026 | *** | -12.677 | *** | -15.322 | *** | -0.055 |  | -3.284 | *** | -8.823 | *** | -11.488 | *** |
|  | 65 | -0.135 |  | -0.222 |  | 1.214 |  | -3.983 |  | -5.508 | * | -5.284 | *** | -3.068 | * | -0.173 |  | -1.306 |  | -0.638 |  |
| active leisure | 10 | 12.755 | ** | 11.491 | *** | 24.111 | ** | 22.102 | *** | 21.128 | * | 14.591 | *** | 18.892 | ** | 15.191 | *** | 33.835 | *** | 33.019 | *** |
|  | 18 | 19.678 | *** | 7.212 | ** | 11.501 | *** | 1.335 |  | 13.190 | ** | 8.878 | *** | -3.165 |  | 11.924 | *** | 11.799 | ** | 27.391 | *** |
|  | 30 | 14.273 | *** | 9.320 | *** | 10.036 | *** | 3.532 | * | 5.737 | ** | 5.552 | *** | 5.549 |  | 4.473 | * | 6.968 | ** | 16.767 | *** |
|  | 45 | 24.153 | *** | 28.632 | *** | 20.199 | *** | 3.757 | * | 7.015 | *** | 8.547 | *** | -3.142 |  | 5.645 | ** | 5.363 | * | 20.773 | *** |
|  | 65 | 64.393 | *** | 61.861 | *** | 42.656 | *** | 27.071 | *** | 8.400 | *** | 28.926 | *** | 21.422 | *** | 25.558 | *** | 12.890 | *** | 28.402 | *** |
| screen-based leisure | 10 | 24.949 | *** | 23.016 | *** | 23.177 | * | 49.166 | *** | 36.918 | ** | 18.622 | *** | 34.841 | ** | 65.915 | *** | 65.296 | *** | 4.795 |  |
|  | 18 | 37.766 | *** | 12.226 | *** | 31.994 | *** | 31.517 | *** | 37.058 | *** | 39.144 | *** | 59.953 | *** | 43.568 | *** | 44.956 | *** | 13.530 |  |
|  | 30 | 35.507 | *** | 29.615 | *** | 37.386 | *** | 19.664 | *** | 38.466 | *** | 18.807 | *** | 48.640 | *** | 34.622 | *** | 30.297 | *** | 28.960 | *** |
|  | 45 | 29.419 | *** | 32.661 | *** | 26.324 | *** | 23.892 | *** | 34.921 | *** | 12.023 | *** | 51.859 | *** | 48.908 | *** | 34.933 | *** | 28.814 | *** |
|  | 65 | 15.179 | ** | 37.683 | *** | 20.336 | *** | 40.254 | *** | 34.364 | *** | 36.229 | *** | 31.108 | ** | 40.149 | *** | 42.859 | *** | 48.683 | *** |
| other lesiure | 10 | -4.460 |  | -5.691 |  | -15.376 |  | -32.741 | *** | -15.023 |  | -14.182 | *** | -43.054 | *** | -25.098 | *** | -17.500 |  | 35.445 | * |
|  | 18 | 10.468 |  | 24.117 | *** | -4.332 |  | -2.913 |  | 10.270 |  | 9.871 | ** | -13.248 |  | -16.612 | ** | 6.447 |  | 26.698 | ** |
|  | 30 | 7.554 | * | 18.815 | *** | -10.760 | *** | -21.380 | *** | 3.490 |  | 14.654 | *** | -6.841 |  | -17.912 | *** | -12.850 | * | -2.440 |  |
|  | 45 | 16.253 | *** | 21.340 | *** | -4.181 |  | -20.753 | *** | 11.524 | ** | 9.070 | *** | -14.300 | * | -20.415 | *** | -29.700 | *** | -21.467 | *** |
|  | 65 | 34.639 | *** | 31.637 | *** | 7.916 | * | -18.377 | * | 16.384 | * | -1.347 |  | -15.176 |  | -20.946 | *** | -36.301 | *** | -23.602 | ** |

Notes: OLS regressions control for day of the week. *** p<0.001, ** p<0.01, * p<0.05

Table A4. Coefficient for Category Men (ref=Women) from the OLS regressions by country and age

|  |  | ES | | IT | | FR | | NL | | HU | | KR | | FI | | UK | | CA | | US | |
| --- | --- | --- | --- | --- | --- | --- | --- | --- | --- | --- | --- | --- | --- | --- | --- | --- | --- | --- | --- | --- | --- |
| ACT | AGE | coef. Men | sig | coef. Men | sig | coef. Men | sig | coef. Men | sig | coef. Men | sig | coef. Men | sig | coef. Men | sig | coef. Men | sig | coef. Men | sig | coef. Men | sig |
| sleep | 10 | 13.423 | * | 5.201 |  | 3.026 |  | 6.824 |  | -7.664 |  | 8.146 | *** | 8.223 |  | -1.213 |  | -13.186 |  | -10.128 |  |
|  | 18 | 3.794 |  | 0.580 |  | -7.662 |  | -19.164 | *** | -13.508 |  | -7.669 | ** | -5.539 |  | 1.047 |  | -15.393 | * | -19.324 | * |
|  | 30 | 0.341 |  | 0.557 |  | -13.831 | *** | -21.823 | *** | -6.317 |  | 13.140 | *** | -12.112 | * | -4.853 |  | -17.134 | *** | -15.855 | ** |
|  | 45 | 17.093 | *** | 7.033 | ** | -10.342 | *** | -7.129 | ** | 2.102 |  | 19.092 | *** | -1.921 |  | -14.768 | *** | -11.033 | *** | -7.106 |  |
|  | 65 | 27.105 | *** | 11.315 | ** | 2.248 |  | 2.485 |  | 6.665 |  | 14.429 | *** | 5.004 |  | -0.982 |  | -3.033 |  | 4.865 |  |
| personal care | 10 | -12.318 | *** | -6.734 | *** | -14.561 | *** | -20.470 | *** | -7.651 | * | -7.235 | *** | -17.068 | *** | -19.062 | *** | -21.994 | *** | -12.322 | * |
|  | 18 | -11.500 | *** | -10.414 | *** | -12.146 | *** | -11.551 | *** | -0.606 |  | -14.394 | *** | -16.359 | *** | -20.669 | *** | -13.526 | *** | -15.113 | *** |
|  | 30 | -1.727 |  | -1.351 |  | -9.591 | *** | -4.368 | ** | 5.228 | * | -3.772 | *** | -12.517 | *** | -13.541 | *** | -11.354 | *** | -11.626 | *** |
|  | 45 | -1.289 |  | 2.078 | ** | -8.313 | *** | -5.789 | *** | 1.441 |  | -1.350 |  | -7.319 | *** | -12.001 | *** | -12.633 | *** | -15.890 | *** |
|  | 65 | -1.971 |  | 6.551 | *** | -4.297 | ** | -2.059 |  | 13.112 | *** | 3.613 | ** | 6.266 | * | -5.820 | ** | -12.939 | *** | -17.035 | *** |
| meals | 10 | -0.015 |  | 4.435 | * | 4.967 |  | -1.362 |  | -1.843 |  | 2.260 | ** | 0.221 |  | -0.268 |  | 7.247 | * | -2.842 |  |
|  | 18 | -3.306 |  | 0.712 |  | 2.262 |  | 1.068 |  | 7.291 | * | -3.143 | ** | 0.357 |  | 8.524 | ** | 5.232 | * | 4.497 |  |
|  | 30 | 0.808 |  | 3.470 | ** | 2.063 |  | -1.530 |  | 5.225 | * | 0.506 |  | -0.056 |  | 0.015 |  | 3.023 |  | 3.816 | * |
|  | 45 | 2.609 | * | 4.896 | *** | 5.104 | *** | -3.386 | * | 8.452 | *** | 3.750 | *** | 0.057 |  | 2.233 |  | 5.481 | *** | 5.277 | ** |
|  | 65 | 3.186 | * | 5.623 | *** | 1.408 |  | 7.229 | * | 11.952 | *** | 2.102 |  | 0.185 |  | 2.031 |  | 2.515 |  | 1.759 |  |
| paid work | 10 | 0.159 |  | 3.398 | * | -4.066 |  | 7.389 |  | 8.905 | ** | -0.109 |  | -4.657 |  | 7.203 |  | -1.065 |  | -1.489 |  |
|  | 18 | 22.431 | ** | 30.655 | *** | 47.630 | *** | 53.972 | *** | 48.414 | *** | 28.407 | *** | 55.919 | *** | 48.602 | *** | 30.913 | ** | 17.408 |  |
|  | 30 | 56.620 | *** | 91.694 | *** | 93.192 | *** | 139.362 | *** | 73.166 | *** | 66.532 | *** | 62.468 | *** | 89.442 | *** | 65.015 | *** | 54.376 | *** |
|  | 45 | 40.114 | *** | 54.353 | *** | 52.763 | *** | 88.187 | *** | 55.479 | *** | 45.724 | *** | 25.284 | ** | 51.392 | *** | 38.972 | *** | 40.948 | *** |
|  | 65 | -0.076 |  | 2.151 |  | 4.227 | ** | 8.536 | *** | 28.705 | *** | 9.689 | * | 4.749 |  | 16.651 | *** | 4.160 |  | 6.762 |  |
| study | 10 | -14.403 |  | -12.881 |  | -6.296 |  | -1.779 |  | -6.870 |  | -12.536 | ** | 34.389 | * | -5.194 |  | -26.213 |  | -26.986 |  |
|  | 18 | -3.233 |  | 0.611 |  | -12.909 | * | 6.904 |  | -18.302 |  | 11.594 | * | -22.833 | * | 11.073 |  | -6.539 |  | 4.264 |  |
|  | 30 | -0.878 |  | 1.526 |  | -1.274 |  | 0.803 |  | 1.427 |  | 5.450 | ** | 0.197 |  | 2.832 |  | 2.907 |  | -3.451 |  |
|  | 45 | -0.205 |  | -0.879 | * | -0.820 | * | 3.400 | * | -0.435 |  | -0.615 |  | -2.673 |  | -0.458 |  | 0.738 |  | -1.754 |  |
|  | 65 | -0.535 |  | 0.324 |  | -1.288 | ** | 4.731 | ** | 0.041 |  | -2.954 | * | 0.370 |  | -0.382 |  | 0.233 |  | 0.960 |  |
| housework | 10 | -22.124 | *** | -23.366 | *** | -27.362 | *** | -20.786 | *** | -27.546 | *** | -7.901 | *** | -25.537 | *** | -23.253 | *** | -15.521 | * | -19.457 | * |
|  | 18 | -52.488 | *** | -72.875 | *** | -42.985 | *** | -44.927 | *** | -62.908 | *** | -48.483 | *** | -23.219 | ** | -51.755 | *** | -36.691 | *** | -35.569 | *** |
|  | 30 | -99.646 | *** | -147.941 | *** | -72.535 | *** | -80.665 | *** | -121.377 | *** | -122.337 | *** | -59.657 | *** | -75.603 | *** | -54.677 | *** | -59.334 | *** |
|  | 45 | -162.907 | *** | -203.780 | *** | -84.567 | *** | -95.875 | *** | -127.747 | *** | -141.470 | *** | -54.756 | *** | -77.596 | *** | -51.685 | *** | -61.711 | *** |
|  | 65 | -160.648 | *** | -195.577 | *** | -94.618 | *** | -73.691 | *** | -118.335 | *** | -136.419 | *** | -72.002 | *** | -67.033 | *** | -42.606 | *** | -71.957 | *** |
| care for tohers | 10 | 0.865 |  | -1.073 |  | 4.385 |  | -6.484 | *** | -1.922 |  | -0.640 |  | -2.245 | * | -1.184 | ** | -8.134 |  | -6.991 | * |
|  | 18 | -15.851 | *** | -8.410 | *** | -16.158 | *** | -11.780 | *** | -21.173 | *** | -19.741 | *** | -20.051 | *** | -29.565 | *** | -26.600 | *** | -18.772 | *** |
|  | 30 | -32.031 | *** | -30.955 | *** | -33.385 | *** | -31.158 | *** | -27.918 | *** | -48.870 | *** | -27.582 | *** | -34.833 | *** | -33.182 | *** | -26.648 | *** |
|  | 45 | -11.332 | *** | -8.016 | *** | -5.680 | *** | -9.977 | *** | -12.714 | *** | -12.246 | *** | -1.870 |  | -3.832 | *** | -9.491 | *** | -10.921 | *** |
|  | 65 | -3.648 |  | -2.852 |  | -0.678 |  | 5.079 | * | 5.341 | * | -6.841 | *** | 4.168 | ** | -0.407 |  | -2.687 |  | -1.725 |  |
| active leisure | 10 | 12.791 | ** | 11.366 | *** | 24.881 | ** | 22.363 | *** | 21.445 | * | 14.592 | *** | 18.738 | ** | 14.895 | *** | 33.577 | *** | 33.085 | *** |
|  | 18 | 19.430 | *** | 8.798 | *** | 12.996 | *** | -0.025 |  | 11.267 | ** | 8.542 | *** | -0.850 |  | 12.061 | *** | 11.681 | ** | 24.636 | *** |
|  | 30 | 16.192 | *** | 13.052 | *** | 10.139 | *** | 3.244 |  | 7.259 | ** | 12.347 | *** | 5.835 |  | 4.287 | * | 7.476 | ** | 18.413 | *** |
|  | 45 | 34.608 | *** | 40.222 | *** | 20.349 | *** | 5.791 | ** | 8.236 | *** | 20.911 | *** | -1.968 |  | 6.379 | ** | 6.972 | ** | 20.986 | *** |
|  | 65 | 65.462 | *** | 62.411 | *** | 43.160 | *** | 26.573 | *** | 8.499 | *** | 32.271 | *** | 22.850 | *** | 22.853 | *** | 11.479 | *** | 25.085 | *** |
| screen-based leisure | 10 | 24.905 | *** | 23.554 | *** | 25.263 | * | 49.439 | *** | 36.505 | ** | 18.580 | *** | 35.362 | ** | 64.661 | *** | 62.289 | *** | 5.091 |  |
|  | 18 | 32.448 | *** | 17.463 | *** | 29.798 | *** | 35.513 | *** | 36.806 | *** | 37.515 | *** | 51.434 | *** | 44.672 | *** | 42.408 | *** | 8.031 |  |
|  | 30 | 41.868 | *** | 37.039 | *** | 31.877 | *** | 21.850 | *** | 42.914 | *** | 46.287 | *** | 47.818 | *** | 40.108 | *** | 35.215 | *** | 33.410 | *** |
|  | 45 | 47.761 | *** | 46.010 | *** | 29.670 | *** | 30.451 | *** | 41.077 | *** | 46.503 | *** | 52.640 | *** | 54.228 | *** | 46.720 | *** | 38.405 | *** |
|  | 65 | 21.413 | *** | 44.452 | *** | 28.147 | *** | 39.311 | *** | 36.347 | *** | 70.401 | *** | 33.787 | *** | 51.195 | *** | 51.654 | *** | 68.175 | *** |
| other lesiure | 10 | -4.423 |  | -5.513 |  | -16.464 |  | -33.912 | *** | -15.474 |  | -14.190 | *** | -42.260 | *** | -24.686 | *** | -18.985 |  | 35.326 | * |
|  | 18 | 5.447 |  | 23.509 | *** | -4.003 |  | -6.157 |  | 4.820 |  | 5.147 |  | -13.902 |  | -16.580 | ** | 2.990 |  | 22.531 | * |
|  | 30 | 9.582 | ** | 24.168 | *** | -10.850 | *** | -20.351 | *** | 9.325 |  | 20.228 | *** | -1.892 |  | -12.239 | ** | -5.854 |  | 4.166 |  |
|  | 45 | 24.653 | *** | 40.038 | *** | -2.757 |  | -7.855 |  | 18.031 | *** | 16.061 | *** | -11.369 | * | -13.867 | ** | -20.718 | *** | -15.768 | ** |
|  | 65 | 39.335 | *** | 44.331 | *** | 15.090 | *** | 8.087 |  | 17.582 | * | 15.622 | *** | 3.722 |  | -16.617 | ** | -14.457 | * | -19.368 | * |

Notes: OLS regressions control for day of the week (as in Table A3), but also for educational attainment, employment status, children in the household and union status. *** p<0.001, ** p<0.01, * p<0.05

Table A5. Median and Interquartile Range by activity. Men’s daily minutes by country

| **SEX** | **ACTIVITY** | **MEASURE** | **Spain** | **Italy** | **France** | **Netherlands** | **Hungary** | **South Korea** | **Finland** | **UK** | **Canada** | **US** |
| --- | --- | --- | --- | --- | --- | --- | --- | --- | --- | --- | --- | --- |
| Men | Sleeping | Median | 520 | 520 | 500 | 495 | 510 | 470 | 520 | 520 | 490 | 510 |
|  |  | p25 | 450 | 450 | 450 | 435 | 445 | 420 | 460 | 450 | 420 | 440 |
|  |  | p75 | 600 | 590 | 570 | 570 | 580 | 540 | 600 | 590 | 560 | 600 |
|  | Personal care | Median | 40 | 50 | 40 | 45 | 70 | 70 | 40 | 40 | 30 | 30 |
|  |  | p25 | 30 | 30 | 30 | 30 | 50 | 50 | 20 | 20 | 10 | 5 |
|  |  | p75 | 60 | 70 | 60 | 60 | 95 | 90 | 60 | 70 | 45 | 60 |
|  | Meals | Median | 90 | 110 | 130 | 75 | 90 | 60 | 80 | 80 | 60 | 45 |
|  |  | p25 | 70 | 80 | 90 | 45 | 70 | 40 | 50 | 40 | 25 | 25 |
|  |  | p75 | 130 | 140 | 170 | 105 | 120 | 90 | 100 | 120 | 90 | 75 |
|  | Paid work | Median | 0 | 0 | 0 | 0 | 60 | 90 | 0 | 0 | 0 | 0 |
|  |  | p25 | 0 | 0 | 0 | 0 | 0 | 0 | 0 | 0 | 0 | 0 |
|  |  | p75 | 420 | 360 | 360 | 495 | 440 | 460 | 160 | 360 | 475 | 455 |
|  | Study | Median | 0 | 0 | 0 | 0 | 0 | 0 | 0 | 0 | 0 | 0 |
|  |  | p25 | 0 | 0 | 0 | 0 | 0 | 0 | 0 | 0 | 0 | 0 |
|  |  | p75 | 0 | 0 | 0 | 0 | 0 | 0 | 0 | 0 | 0 | 0 |
|  | Housework | Median | 40 | 30 | 90 | 45 | 60 | 0 | 80 | 70 | 70 | 45 |
|  |  | p25 | 0 | 0 | 20 | 0 | 10 | 0 | 30 | 20 | 15 | 2 |
|  |  | p75 | 120 | 110 | 180 | 135 | 150 | 40 | 170 | 160 | 180 | 125 |
|  | Care for others | Median | 0 | 0 | 0 | 0 | 0 | 0 | 0 | 0 | 0 | 0 |
|  |  | p25 | 0 | 0 | 0 | 0 | 0 | 0 | 0 | 0 | 0 | 0 |
|  |  | p75 | 0 | 0 | 0 | 0 | 0 | 0 | 0 | 0 | 0 | 15 |
|  | Active leisure | Median | 30 | 0 | 0 | 0 | 0 | 0 | 0 | 0 | 0 | 0 |
|  |  | p25 | 0 | 0 | 0 | 0 | 0 | 0 | 0 | 0 | 0 | 0 |
|  |  | p75 | 120 | 110 | 90 | 60 | 0 | 60 | 90 | 60 | 45 | 60 |
|  | Screen-based leisure | Median | 150 | 120 | 140 | 105 | 170 | 130 | 180 | 180 | 145 | 150 |
|  |  | p25 | 70 | 60 | 60 | 30 | 90 | 60 | 90 | 80 | 60 | 60 |
|  |  | p75 | 260 | 210 | 240 | 195 | 260 | 230 | 290 | 300 | 260 | 300 |
|  | Other leisure | Median | 100 | 160 | 90 | 150 | 90 | 140 | 130 | 90 | 118 | 95 |
|  |  | p25 | 30 | 70 | 20 | 60 | 25 | 90 | 50 | 20 | 30 | 15 |
|  |  | p75 | 200 | 270 | 200 | 270 | 180 | 220 | 260 | 200 | 265 | 230 |
|  | Travel | Median | 60 | 70 | 60 | 60 | 50 | 80 | 50 | 60 | 60 | 55 |
|  |  | p25 | 20 | 40 | 20 | 30 | 20 | 50 | 10 | 20 | 20 | 20 |
|  |  | p75 | 100 | 120 | 110 | 105 | 90 | 120 | 90 | 110 | 100 | 100 |
|  | Other | Median | 0 | 0 | 0 | 0 | 0 | 0 | 0 | 0 | 0 | 0 |
|  |  | p25 | 0 | 0 | 0 | 0 | 0 | 0 | 0 | 0 | 0 | 0 |
|  |  | p75 | 0 | 0 | 0 | 0 | 0 | 0 | 0 | 10 | 0 | 0 |

Source: Multinational Time Use Study [32].

Table A5 (Continuation). Median and Interquartile Range by activity. Women’s daily minutes by country

| **SEX** | **ACTIVITY** | **MEASURE** | **Spain** | **Italy** | **France** | **Netherlands** | **Hungary** | **South Korea** | **Finland** | **UK** | **Canada** | **US** |
| --- | --- | --- | --- | --- | --- | --- | --- | --- | --- | --- | --- | --- |
| Women | Sleeping | Median | 510 | 520 | 510 | 510 | 510 | 470 | 530 | 520 | 510 | 530 |
|  |  | p25 | 450 | 460 | 450 | 465 | 450 | 420 | 470 | 460 | 450 | 450 |
|  |  | p75 | 590 | 580 | 570 | 570 | 570 | 540 | 600 | 590 | 570 | 603 |
|  | Personal care | Median | 50 | 50 | 50 | 45 | 65 | 70 | 50 | 60 | 40 | 45 |
|  |  | p25 | 30 | 30 | 30 | 30 | 45 | 50 | 30 | 40 | 20 | 20 |
|  |  | p75 | 70 | 80 | 70 | 75 | 90 | 90 | 70 | 80 | 60 | 75 |
|  | Meals | Median | 100 | 110 | 120 | 75 | 90 | 70 | 70 | 80 | 50 | 45 |
|  |  | p25 | 70 | 80 | 90 | 45 | 70 | 40 | 50 | 40 | 20 | 20 |
|  |  | p75 | 120 | 140 | 170 | 105 | 110 | 90 | 100 | 120 | 90 | 75 |
|  | Paid work | Median | 0 | 0 | 0 | 0 | 0 | 0 | 0 | 0 | 0 | 0 |
|  |  | p25 | 0 | 0 | 0 | 0 | 0 | 0 | 0 | 0 | 0 | 0 |
|  |  | p75 | 0 | 0 | 90 | 195 | 240 | 290 | 10 | 0 | 375 | 225 |
|  | Study | Median | 0 | 0 | 0 | 0 | 0 | 0 | 0 | 0 | 0 | 0 |
|  |  | p25 | 0 | 0 | 0 | 0 | 0 | 0 | 0 | 0 | 0 | 0 |
|  |  | p75 | 0 | 0 | 0 | 0 | 0 | 0 | 0 | 0 | 0 | 0 |
|  | Housework | Median | 190 | 230 | 180 | 135 | 210 | 140 | 140 | 150 | 140 | 120 |
|  |  | p25 | 90 | 100 | 90 | 45 | 110 | 40 | 70 | 70 | 60 | 40 |
|  |  | p75 | 310 | 370 | 280 | 255 | 315 | 240 | 240 | 260 | 260 | 235 |
|  | Care for others | Median | 0 | 0 | 0 | 0 | 0 | 0 | 0 | 0 | 0 | 0 |
|  |  | p25 | 0 | 0 | 0 | 0 | 0 | 0 | 0 | 0 | 0 | 0 |
|  |  | p75 | 40 | 10 | 30 | 30 | 20 | 30 | 0 | 10 | 9 | 60 |
|  | Active leisure | Median | 0 | 0 | 0 | 0 | 0 | 0 | 20 | 0 | 0 | 0 |
|  |  | p25 | 0 | 0 | 0 | 0 | 0 | 0 | 0 | 0 | 0 | 0 |
|  |  | p75 | 80 | 60 | 60 | 30 | 0 | 40 | 70 | 40 | 30 | 20 |
|  | Screen-based leisure | Median | 120 | 90 | 120 | 75 | 135 | 110 | 130 | 140 | 110 | 120 |
|  |  | p25 | 60 | 30 | 40 | 15 | 75 | 50 | 60 | 60 | 30 | 40 |
|  |  | p75 | 220 | 160 | 200 | 150 | 220 | 200 | 220 | 230 | 210 | 245 |
|  | Other leisure | Median | 90 | 140 | 110 | 180 | 90 | 130 | 160 | 120 | 150 | 120 |
|  |  | p25 | 20 | 60 | 30 | 75 | 20 | 80 | 70 | 40 | 45 | 30 |
|  |  | p75 | 180 | 230 | 210 | 285 | 180 | 210 | 270 | 230 | 300 | 245 |
|  | Travel | Median | 50 | 50 | 60 | 60 | 40 | 70 | 50 | 50 | 50 | 51 |
|  |  | p25 | 20 | 20 | 10 | 30 | 10 | 40 | 10 | 20 | 20 | 20 |
|  |  | p75 | 90 | 100 | 100 | 105 | 80 | 120 | 90 | 100 | 95 | 100 |
|  | Other | Median | 0 | 0 | 0 | 0 | 0 | 0 | 0 | 0 | 0 | 0 |
|  |  | p25 | 0 | 0 | 0 | 0 | 0 | 0 | 0 | 0 | 0 | 0 |
|  |  | p75 | 0 | 0 | 0 | 0 | 0 | 0 | 0 | 10 | 0 | 0 |

Source: Multinational Time Use Study [32].

Table A6. Net differences between retired and other employment status.

|  | Sleeping | Personal care | Meals | Paid work | Study | Housework | Care for others | Active leisure | Screen-based leisure | Other leisure |
| --- | --- | --- | --- | --- | --- | --- | --- | --- | --- | --- |
| AGE GROUP: 45 to 64 |  |  |  |  |  |  |  |  |  |  |
| Employed (ref. retired) | -31,560*** | 0.125 | -12,814*** | 300,678*** | -2,826*** | -76,538*** | -13,545*** | -39,491*** | -62,949*** | -60,318*** |
|  | (1,526) | (0,615) | (0,791) | (1,286) | (0,404) | (1,993) | (0,931) | (1,372) | (1,917) | (1,972) |
| Non employed (ref. retired) | 0.51 | 0.934 | -2,412** | 26,424*** | 1,388** | 11,951*** | 1.249 | -20,968*** | -0.535 | -16,444*** |
|  | (1,691) | (0,711) | (0,859) | (1,232) | (0,516) | (2,244) | (1,065) | (1,443) | (2,155) | (2,157) |
| Constant | 503,158*** | 44,057*** | 69,893*** | -14,627*** | 18,891*** | 240,698*** | 32,539*** | 56,064*** | 180,406*** | 92,667*** |
|  | (8,869) | (2,956) | (3,984) | (2,358) | (2,629) | (9,608) | (3,835) | (5,246) | (10,43) | (9,571) |
| Observations | 63,611 | 63,611 | 63,611 | 201,972 | 63,611 | 63,611 | 63,611 | 63,611 | 63,611 | 63,611 |
| R-squared | 0.079 | 0.069 | 0.198 | 0.481 | 0.018 | 0.316 | 0.064 | 0.074 | 0.125 | 0.124 |
| AGE GROUP: 65 or older |  |  |  |  |  |  |  |  |  |  |
| Employed (ref. retired) | -23,483*** | -2,339** | -7,779*** | 251,016*** | -1,966*** | -49,316*** | -7,045*** | -31,608*** | -75,702*** | -58,928*** |
|  | (2,154) | (0,899) | (1,105) | (4,35) | (0,43) | (2,629) | (0,835) | (1,772) | (2,798) | (2,822) |
| Non employed (ref. retired) | 7,740*** | 1.43 | -0.073 | 7,576*** | -0,548** | 14,230*** | -1.332 | -15,744*** | 2.493 | -9,601*** |
|  | (2,273) | (0,955) | (0,907) | (1,178) | (0,207) | (2,671) | (1,083) | (1,338) | (2,536) | (2,475) |
| Constant | 276,628*** | 38,083*** | 58,074*** | 49,043*** | 9,400*** | 468,358*** | 64,943*** | 161,083*** | 106,807*** | 24,088* |
|  | (11,661) | (3,986) | (5,02) | (6,091) | (1,482) | (11,605) | (4,228) | (7,401) | (12,656) | (12,217) |
| Observations | 37,885 | 37,885 | 37,885 | 37,885 | 37,885 | 37,885 | 37,885 | 37,885 | 37,885 | 37,885 |
| R-squared | 0.105 | 0.038 | 0.172 | 0.494 | 0.022 | 0.233 | 0.025 | 0.103 | 0.091 | 0.092 |

Source: Multinational Time Use Study [32]. OLS also control by country, continuous age, educational attainment, employment status, children in the household, union status and day of the week. *** p<0.001, ** p<0.01, * p<0.05

Table A7 Median by activity for those who spend at least 1 minute. Men’s and Women’s daily minutes by country

|  |  | **Spain** | | **Italy** | | **France** | | **Netherlands** | | **Hungary** | | **South Korea** | | **Finland** | | **UK** | | **Canada** | | **US** | |
| --- | --- | --- | --- | --- | --- | --- | --- | --- | --- | --- | --- | --- | --- | --- | --- | --- | --- | --- | --- | --- | --- |
| AGE | ACTIVITY | M | W | M | W | M | W | M | W | M | W | M | W | M | W | M | W | M | W | M | W |
| 10-17 | Sleeping | 570 | 560 | 560 | 560 | 600 | 590 | 555 | 540 | 570 | 573 | 490 | 490 | 600 | 585 | 600 | 600 | 540 | 554 | 570 | 575 |
|  | Personal care | 40 | 50 | 50 | 50 | 40 | 60 | 45 | 60 | 55 | 65 | 70 | 70 | 40 | 50 | 40 | 60 | 30 | 60 | 40 | 60 |
|  | Meals | 90 | 90 | 100 | 100 | 110 | 110 | 60 | 60 | 90 | 85 | 60 | 60 | 70 | 60 | 70 | 70 | 55 | 50 | 40 | 45 |
|  | Paid work | 430 | 385 | 435 | 225 | 110 | 65 | 180 | 180 | 50 | 40 | 165 | 240 | 140 | 175 | 180 | 190 | 310 | 320 | 245 | 232 |
|  | Study | 390 | 385 | 360 | 350 | 350 | 320 | 390 | 390 | 378 | 400 | 470 | 480 | 320 | 300 | 350 | 340 | 370 | 375 | 397 | 368 |
|  | Housework | 40 | 50 | 30 | 60 | 60 | 60 | 30 | 45 | 45 | 60 | 20 | 30 | 40 | 50 | 30 | 50 | 45 | 50 | 40 | 60 |
|  | Care for others | 60 | 30 | 30 | 30 | 90 | 40 | 45 | 60 | 60 | 80 | 30 | 30 | 20 | 30 | 30 | 30 | 48 | 104 | 10 | 27 |
|  | Active leisure | 110 | 110 | 120 | 100 | 120 | 110 | 120 | 90 | 90 | 85 | 50 | 40 | 100 | 70 | 90 | 70 | 127 | 95 | 120 | 90 |
|  | Screen-based leisure | 170 | 150 | 150 | 130 | 210 | 180 | 150 | 105 | 182 | 150 | 140 | 130 | 220 | 190 | 235 | 170 | 180 | 148 | 151 | 175 |
|  | Other leisure | 140 | 150 | 170 | 170 | 120 | 140 | 150 | 165 | 113 | 130 | 110 | 120 | 180 | 190 | 130 | 160 | 150 | 175 | 180 | 180 |
| 18-29 | Sleeping | 520 | 520 | 510 | 510 | 510 | 520 | 495 | 510 | 510 | 530 | 470 | 480 | 540 | 540 | 540 | 540 | 510 | 530 | 540 | 550 |
|  | Personal care | 40 | 50 | 50 | 60 | 40 | 50 | 45 | 60 | 70 | 70 | 70 | 80 | 40 | 50 | 40 | 60 | 30 | 50 | 40 | 60 |
|  | Meals | 90 | 90 | 90 | 90 | 110 | 110 | 60 | 60 | 85 | 80 | 50 | 60 | 60 | 60 | 70 | 70 | 50 | 45 | 45 | 45 |
|  | Paid work | 480 | 430 | 490 | 440 | 470 | 420 | 510 | 480 | 450 | 440 | 450 | 420 | 450 | 410 | 480 | 450 | 485 | 464 | 469 | 450 |
|  | Study | 290 | 300 | 310 | 290 | 330 | 300 | 285 | 225 | 343 | 330 | 320 | 270 | 220 | 225 | 300 | 260 | 340 | 305 | 243 | 180 |
|  | Housework | 60 | 110 | 50 | 110 | 70 | 120 | 60 | 105 | 55 | 130 | 40 | 80 | 70 | 90 | 60 | 110 | 60 | 115 | 60 | 113 |
|  | Care for others | 80 | 150 | 60 | 110 | 60 | 110 | 60 | 150 | 70 | 210 | 40 | 160 | 70 | 160 | 50 | 130 | 70 | 168 | 45 | 95 |
|  | Active leisure | 110 | 80 | 110 | 80 | 110 | 80 | 90 | 90 | 80 | 60 | 70 | 60 | 80 | 70 | 80 | 60 | 105 | 60 | 90 | 55 |
|  | Screen-based leisure | 160 | 130 | 120 | 110 | 170 | 140 | 135 | 90 | 174 | 135 | 160 | 130 | 200 | 150 | 180 | 140 | 150 | 120 | 180 | 140 |
|  | Other leisure | 150 | 140 | 210 | 190 | 130 | 130 | 180 | 180 | 120 | 110 | 150 | 140 | 130 | 140 | 110 | 120 | 155 | 160 | 175 | 150 |
| 30-44 | Sleeping | 480 | 500 | 500 | 500 | 480 | 510 | 465 | 495 | 480 | 490 | 460 | 460 | 500 | 520 | 510 | 510 | 480 | 495 | 502 | 518 |
|  | Personal care | 40 | 40 | 50 | 50 | 40 | 50 | 45 | 45 | 70 | 60 | 60 | 60 | 40 | 50 | 40 | 60 | 30 | 45 | 40 | 50 |
|  | Meals | 90 | 90 | 100 | 100 | 120 | 110 | 75 | 75 | 90 | 90 | 60 | 70 | 70 | 70 | 70 | 70 | 60 | 60 | 50 | 45 |
|  | Paid work | 490 | 420 | 490 | 410 | 480 | 420 | 510 | 420 | 470 | 440 | 470 | 400 | 480 | 450 | 480 | 420 | 500 | 455 | 481 | 450 |
|  | Study | 160 | 150 | 230 | 170 | 90 | 60 | 90 | 90 | 155 | 120 | 110 | 110 | 180 | 150 | 120 | 90 | 235 | 153 | 210 | 142 |
|  | Housework | 90 | 190 | 70 | 240 | 100 | 170 | 75 | 180 | 75 | 210 | 40 | 190 | 90 | 160 | 80 | 170 | 90 | 155 | 75 | 150 |
|  | Care for others | 90 | 120 | 80 | 100 | 60 | 90 | 60 | 105 | 90 | 125 | 50 | 80 | 70 | 70 | 70 | 100 | 85 | 125 | 75 | 103 |
|  | Active leisure | 90 | 80 | 100 | 90 | 90 | 80 | 75 | 60 | 75 | 75 | 70 | 70 | 80 | 70 | 70 | 70 | 90 | 60 | 90 | 60 |
|  | Screen-based leisure | 130 | 100 | 120 | 90 | 150 | 120 | 113 | 105 | 150 | 120 | 120 | 110 | 170 | 120 | 160 | 120 | 130 | 105 | 155 | 125 |
|  | Other leisure | 100 | 100 | 150 | 130 | 110 | 110 | 135 | 150 | 80 | 85 | 140 | 120 | 110 | 120 | 100 | 120 | 120 | 125 | 120 | 120 |
| 45-64 | Sleeping | 500 | 490 | 490 | 500 | 490 | 500 | 480 | 510 | 495 | 500 | 460 | 460 | 510 | 510 | 490 | 510 | 480 | 490 | 507 | 510 |
|  | Personal care | 40 | 50 | 50 | 50 | 40 | 50 | 45 | 60 | 70 | 65 | 70 | 70 | 40 | 50 | 40 | 60 | 30 | 45 | 40 | 60 |
|  | Meals | 100 | 100 | 110 | 110 | 130 | 120 | 75 | 90 | 95 | 90 | 70 | 70 | 80 | 80 | 80 | 80 | 60 | 60 | 50 | 50 |
|  | Paid work | 490 | 420 | 480 | 390 | 470 | 420 | 495 | 375 | 400 | 360 | 450 | 400 | 470 | 450 | 475 | 420 | 495 | 465 | 484 | 455 |
|  | Study | 155 | 110 | 110 | 130 | 60 | 75 | 90 | 75 | 75 | 93 | 80 | 90 | 100 | 170 | 75 | 100 | 143 | 120 | 180 | 160 |
|  | Housework | 100 | 260 | 90 | 320 | 130 | 210 | 90 | 195 | 115 | 241 | 50 | 190 | 110 | 170 | 110 | 190 | 120 | 165 | 85 | 140 |
|  | Care for others | 60 | 80 | 60 | 60 | 60 | 60 | 45 | 60 | 65 | 90 | 30 | 30 | 30 | 30 | 40 | 40 | 85 | 90 | 45 | 60 |
|  | Active leisure | 110 | 90 | 120 | 90 | 120 | 80 | 90 | 75 | 73 | 60 | 80 | 80 | 90 | 70 | 70 | 60 | 90 | 60 | 80 | 58 |
|  | Screen-based leisure | 160 | 140 | 140 | 110 | 160 | 130 | 135 | 120 | 180 | 150 | 130 | 130 | 180 | 140 | 200 | 150 | 177 | 145 | 195 | 166 |
|  | Other leisure | 120 | 120 | 160 | 140 | 120 | 120 | 180 | 210 | 120 | 120 | 150 | 140 | 140 | 170 | 130 | 140 | 150 | 170 | 120 | 150 |
| 65+ | Sleeping | 570 | 560 | 540 | 540 | 520 | 530 | 525 | 540 | 550 | 540 | 500 | 510 | 530 | 540 | 510 | 510 | 510 | 525 | 540 | 540 |
|  | Personal care | 50 | 50 | 50 | 50 | 50 | 60 | 45 | 60 | 75 | 65 | 70 | 60 | 50 | 50 | 50 | 60 | 30 | 45 | 43 | 43 |
|  | Meals | 110 | 100 | 120 | 120 | 140 | 130 | 105 | 90 | 100 | 90 | 90 | 80 | 90 | 90 | 110 | 100 | 80 | 70 | 60 | 60 |
|  | Paid work | 520 | 405 | 430 | 390 | 140 | 60 | 225 | 30 | 160 | 90 | 305 | 210 | 250 | 165 | 260 | 160 | 330 | 368 | 374 | 374 |
|  | Study | 100 | 105 | 120 | 110 | 60 | 70 | 75 | 68 | 215 | 145 | 100 | 100 | 70 | 100 | 80 | 80 | 120 | 99 | 90 | 90 |
|  | Housework | 110 | 250 | 110 | 300 | 140 | 230 | 150 | 225 | 135 | 250 | 60 | 190 | 140 | 210 | 140 | 210 | 140 | 180 | 90 | 90 |
|  | Care for others | 100 | 90 | 70 | 70 | 60 | 60 | 53 | 60 | 90 | 90 | 50 | 40 | 30 | 60 | 30 | 30 | 113 | 75 | 32 | 32 |
|  | Active leisure | 140 | 90 | 130 | 80 | 120 | 80 | 105 | 75 | 60 | 60 | 100 | 70 | 80 | 70 | 90 | 60 | 85 | 60 | 82 | 82 |
|  | Screen-based leisure | 230 | 220 | 180 | 150 | 210 | 180 | 180 | 135 | 220 | 190 | 220 | 180 | 210 | 170 | 240 | 190 | 240 | 210 | 310 | 310 |
|  | Other leisure | 170 | 130 | 220 | 180 | 180 | 180 | 240 | 255 | 170 | 165 | 170 | 170 | 230 | 220 | 170 | 190 | 240 | 270 | 205 | 205 |

Source: Multinational Time Use Study [32].

Table A8. IQR by activity for those who spend at least 1 minute. Men’s and Women’s daily minutes by country

|  |  | **Spain** | | | | **Italy** | | | | **France** | | | | **Netherlands** | | | | **Hungary** | | | |
| --- | --- | --- | --- | --- | --- | --- | --- | --- | --- | --- | --- | --- | --- | --- | --- | --- | --- | --- | --- | --- | --- |
|  |  | M | | W | | M | | W | | M | | W | | M | | W | | M | | W | |
| AGE | ACTIVITY | p25 | p75 | p25 | p75 | p25 | p75 | p25 | p75 | p25 | p75 | p25 | p75 | p25 | p75 | p25 | p75 | p25 | p75 | p25 | p75 |
|  |  |  |  |  |  |  |  |  |  |  |  |  |  |  |  |  |  |  |  |  |  |
| 10-17 | Sleeping | 500 | 640 | 500 | 640 | 500 | 650 | 500 | 640 | 520 | 680 | 530 | 670 | 495 | 615 | 495 | 615 | 510 | 630 | 520 | 640 |
|  | Personal care | 30 | 60 | 40 | 80 | 30 | 70 | 30 | 80 | 30 | 60 | 40 | 80 | 30 | 60 | 30 | 75 | 40 | 75 | 50 | 90 |
|  | Meals | 70 | 120 | 70 | 120 | 80 | 130 | 70 | 130 | 70 | 140 | 80 | 140 | 45 | 90 | 45 | 90 | 70 | 110 | 65 | 110 |
|  | Paid work | 110 | 480 | 250 | 475 | 275 | 490 | 190 | 300 | 60 | 530 | 50 | 255 | 75 | 420 | 75 | 375 | 20 | 170 | 15 | 68 |
|  | Study | 170 | 470 | 150 | 495 | 150 | 440 | 120 | 450 | 120 | 490 | 120 | 480 | 270 | 465 | 270 | 465 | 208 | 450 | 250 | 470 |
|  | Housework | 20 | 70 | 20 | 100 | 20 | 70 | 30 | 110 | 30 | 110 | 30 | 120 | 15 | 60 | 30 | 90 | 15 | 90 | 25 | 135 |
|  | Care for others | 20 | 120 | 20 | 50 | 20 | 80 | 20 | 70 | 45 | 145 | 20 | 70 | 23 | 75 | 30 | 120 | 30 | 120 | 20 | 120 |
|  | Active leisure | 60 | 150 | 60 | 160 | 70 | 170 | 60 | 150 | 70 | 200 | 60 | 180 | 75 | 165 | 60 | 135 | 60 | 145 | 45 | 150 |
|  | Screen-based leisure | 100 | 270 | 90 | 240 | 90 | 230 | 70 | 200 | 110 | 300 | 90 | 270 | 75 | 225 | 60 | 165 | 108 | 285 | 90 | 230 |
|  | Other leisure | 70 | 250 | 70 | 270 | 90 | 290 | 90 | 280 | 60 | 210 | 70 | 240 | 60 | 270 | 90 | 285 | 55 | 190 | 60 | 210 |
| 18-29 | Sleeping | 440 | 610 | 450 | 600 | 440 | 600 | 440 | 590 | 450 | 600 | 460 | 600 | 435 | 570 | 465 | 585 | 435 | 600 | 455 | 600 |
|  | Personal care | 30 | 60 | 30 | 80 | 30 | 80 | 40 | 90 | 30 | 60 | 30 | 80 | 30 | 75 | 45 | 90 | 50 | 97 | 50 | 95 |
|  | Meals | 60 | 120 | 70 | 120 | 60 | 130 | 70 | 130 | 70 | 160 | 80 | 150 | 45 | 90 | 45 | 90 | 70 | 110 | 60 | 105 |
|  | Paid work | 400 | 550 | 300 | 490 | 420 | 540 | 320 | 500 | 340 | 520 | 240 | 500 | 435 | 570 | 345 | 525 | 285 | 515 | 280 | 480 |
|  | Study | 170 | 440 | 150 | 440 | 180 | 440 | 170 | 410 | 125 | 500 | 120 | 450 | 150 | 420 | 120 | 360 | 210 | 435 | 158 | 465 |
|  | Housework | 30 | 130 | 50 | 200 | 20 | 100 | 50 | 220 | 40 | 130 | 60 | 200 | 30 | 120 | 45 | 195 | 20 | 108 | 52 | 225 |
|  | Care for others | 40 | 150 | 70 | 230 | 30 | 150 | 40 | 200 | 40 | 135 | 60 | 190 | 30 | 113 | 90 | 225 | 35 | 130 | 120 | 330 |
|  | Active leisure | 70 | 180 | 50 | 130 | 60 | 160 | 60 | 130 | 60 | 180 | 40 | 130 | 75 | 135 | 60 | 120 | 60 | 125 | 30 | 100 |
|  | Screen-based leisure | 90 | 280 | 80 | 210 | 70 | 200 | 60 | 170 | 100 | 280 | 80 | 220 | 75 | 225 | 45 | 165 | 100 | 280 | 90 | 215 |
|  | Other leisure | 70 | 280 | 60 | 260 | 120 | 340 | 100 | 300 | 60 | 250 | 60 | 240 | 75 | 315 | 90 | 285 | 55 | 210 | 55 | 185 |
| 30-44 | Sleeping | 420 | 570 | 440 | 570 | 430 | 570 | 450 | 560 | 430 | 550 | 450 | 570 | 420 | 525 | 450 | 555 | 420 | 540 | 440 | 550 |
|  | Personal care | 30 | 60 | 30 | 60 | 30 | 70 | 30 | 70 | 30 | 60 | 30 | 70 | 30 | 60 | 30 | 60 | 50 | 95 | 45 | 85 |
|  | Meals | 60 | 120 | 70 | 120 | 70 | 130 | 70 | 130 | 80 | 160 | 80 | 160 | 45 | 90 | 45 | 105 | 70 | 115 | 70 | 110 |
|  | Paid work | 420 | 580 | 300 | 480 | 410 | 560 | 300 | 490 | 350 | 540 | 210 | 490 | 465 | 570 | 270 | 510 | 360 | 540 | 270 | 480 |
|  | Study | 90 | 260 | 80 | 240 | 120 | 370 | 110 | 300 | 40 | 180 | 30 | 180 | 30 | 195 | 45 | 158 | 90 | 250 | 75 | 270 |
|  | Housework | 40 | 160 | 110 | 290 | 30 | 140 | 140 | 360 | 50 | 190 | 90 | 260 | 45 | 150 | 90 | 270 | 30 | 150 | 120 | 320 |
|  | Care for others | 40 | 170 | 60 | 210 | 40 | 140 | 40 | 180 | 30 | 110 | 50 | 160 | 30 | 120 | 60 | 165 | 50 | 150 | 60 | 215 |
|  | Active leisure | 60 | 150 | 50 | 120 | 60 | 170 | 60 | 130 | 60 | 170 | 40 | 130 | 45 | 120 | 45 | 105 | 53 | 130 | 60 | 120 |
|  | Screen-based leisure | 80 | 220 | 60 | 170 | 70 | 190 | 60 | 140 | 90 | 230 | 60 | 180 | 60 | 180 | 60 | 150 | 90 | 240 | 70 | 180 |
|  | Other leisure | 50 | 190 | 50 | 180 | 70 | 260 | 60 | 210 | 50 | 200 | 60 | 190 | 60 | 240 | 75 | 255 | 40 | 150 | 40 | 150 |
| 45-64 | Sleeping | 440 | 570 | 440 | 550 | 440 | 560 | 440 | 550 | 430 | 540 | 450 | 560 | 435 | 540 | 450 | 555 | 435 | 555 | 450 | 555 |
|  | Personal care | 30 | 60 | 30 | 70 | 30 | 70 | 30 | 70 | 30 | 60 | 30 | 70 | 30 | 60 | 30 | 75 | 50 | 93 | 45 | 90 |
|  | Meals | 70 | 130 | 70 | 130 | 80 | 140 | 80 | 140 | 90 | 180 | 90 | 170 | 60 | 105 | 60 | 120 | 75 | 123 | 70 | 115 |
|  | Paid work | 420 | 570 | 330 | 480 | 370 | 550 | 290 | 480 | 300 | 540 | 210 | 490 | 435 | 555 | 225 | 495 | 175 | 505 | 130 | 475 |
|  | Study | 80 | 250 | 70 | 180 | 95 | 170 | 110 | 240 | 40 | 120 | 30 | 130 | 45 | 180 | 45 | 135 | 35 | 295 | 60 | 180 |
|  | Housework | 50 | 180 | 160 | 370 | 40 | 170 | 200 | 430 | 60 | 220 | 120 | 310 | 45 | 180 | 120 | 300 | 50 | 200 | 150 | 345 |
|  | Care for others | 30 | 140 | 40 | 160 | 20 | 120 | 20 | 120 | 30 | 120 | 30 | 120 | 30 | 90 | 30 | 120 | 40 | 115 | 45 | 145 |
|  | Active leisure | 60 | 180 | 60 | 130 | 70 | 200 | 60 | 130 | 60 | 190 | 50 | 130 | 60 | 135 | 45 | 120 | 40 | 120 | 35 | 110 |
|  | Screen-based leisure | 90 | 260 | 80 | 220 | 90 | 220 | 60 | 160 | 100 | 240 | 80 | 210 | 75 | 210 | 60 | 180 | 120 | 270 | 95 | 225 |
|  | Other leisure | 60 | 220 | 60 | 200 | 90 | 270 | 70 | 220 | 60 | 220 | 60 | 210 | 90 | 300 | 120 | 315 | 60 | 195 | 60 | 190 |
| 65+ | Sleeping | 510 | 650 | 500 | 630 | 480 | 620 | 490 | 620 | 480 | 570 | 480 | 580 | 480 | 570 | 495 | 585 | 490 | 610 | 490 | 600 |
|  | Personal care | 30 | 70 | 30 | 70 | 30 | 80 | 30 | 80 | 30 | 80 | 40 | 80 | 30 | 75 | 30 | 75 | 50 | 105 | 45 | 90 |
|  | Meals | 90 | 140 | 80 | 130 | 100 | 150 | 90 | 140 | 110 | 180 | 100 | 170 | 75 | 135 | 60 | 120 | 80 | 130 | 70 | 115 |
|  | Paid work | 350 | 600 | 360 | 475 | 270 | 540 | 280 | 500 | 40 | 390 | 40 | 180 | 105 | 405 | 30 | 60 | 70 | 260 | 50 | 193 |
|  | Study | 70 | 130 | 60 | 145 | 60 | 240 | 90 | 140 | 30 | 130 | 45 | 125 | 45 | 120 | 30 | 120 | 158 | 290 | 100 | 160 |
|  | Housework | 50 | 190 | 170 | 340 | 50 | 190 | 210 | 390 | 80 | 230 | 150 | 320 | 75 | 240 | 135 | 315 | 65 | 210 | 175 | 325 |
|  | Care for others | 50 | 160 | 40 | 160 | 40 | 150 | 30 | 140 | 30 | 120 | 30 | 110 | 30 | 135 | 30 | 165 | 55 | 133 | 50 | 150 |
|  | Active leisure | 90 | 220 | 60 | 140 | 80 | 210 | 60 | 120 | 60 | 180 | 50 | 120 | 60 | 180 | 45 | 120 | 30 | 115 | 35 | 90 |
|  | Screen-based leisure | 150 | 330 | 140 | 310 | 120 | 260 | 90 | 220 | 130 | 300 | 120 | 270 | 120 | 255 | 90 | 225 | 150 | 315 | 120 | 270 |
|  | Other leisure | 90 | 265 | 70 | 220 | 130 | 310 | 110 | 270 | 100 | 280 | 100 | 270 | 150 | 375 | 165 | 375 | 100 | 270 | 100 | 255 |

Source: Multinational Time Use Study [32].

Table A8 (continuation). IQR by activity for those who spend at least 1 minute. Men’s and Women’s daily minutes by country

|  |  | **South Korea** | | | | **Finland** | | | | **UK** | | | | **Canada** | | | | **US** | | | |
| --- | --- | --- | --- | --- | --- | --- | --- | --- | --- | --- | --- | --- | --- | --- | --- | --- | --- | --- | --- | --- | --- |
|  |  | M | | W | | M | | W | | M | | W | | M | | W | | M | | W | |
| AGE | ACTIVITY | p25 | p75 | p25 | p75 | p25 | p75 | p25 | p75 | p25 | p75 | p25 | p75 | p25 | p75 | p25 | p75 | p25 | p75 | p25 | p75 |
|  |  |  |  |  |  |  |  |  |  |  |  |  |  |  |  |  |  |  |  |  |  |
| 10-17 | Sleeping | 440 | 550 | 430 | 550 | 520 | 660 | 520 | 660 | 540 | 670 | 540 | 680 | 477 | 645 | 480 | 653 | 480 | 665 | 485 | 675 |
|  | Personal care | 50 | 90 | 60 | 90 | 20 | 50 | 30 | 70 | 30 | 60 | 40 | 90 | 20 | 50 | 30 | 80 | 25 | 60 | 30 | 90 |
|  | Meals | 40 | 90 | 40 | 80 | 40 | 90 | 40 | 90 | 40 | 100 | 40 | 100 | 30 | 90 | 30 | 85 | 23 | 65 | 25 | 65 |
|  | Paid work | 70 | 300 | 80 | 340 | 60 | 270 | 75 | 295 | 40 | 430 | 35 | 320 | 150 | 460 | 180 | 450 | 120 | 393 | 150 | 450 |
|  | Study | 280 | 590 | 290 | 610 | 210 | 390 | 90 | 380 | 150 | 410 | 150 | 420 | 300 | 440 | 285 | 460 | 285 | 469 | 150 | 480 |
|  | Housework | 10 | 50 | 20 | 60 | 20 | 80 | 30 | 100 | 10 | 70 | 20 | 110 | 20 | 80 | 30 | 110 | 10 | 90 | 20 | 150 |
|  | Care for others | 10 | 50 | 20 | 60 | 10 | 30 | 20 | 60 | 20 | 85 | 20 | 60 | 30 | 60 | 38 | 283 | 4 | 45 | 5 | 61 |
|  | Active leisure | 30 | 90 | 30 | 70 | 50 | 170 | 40 | 120 | 60 | 150 | 40 | 120 | 90 | 180 | 60 | 175 | 63 | 228 | 45 | 160 |
|  | Screen-based leisure | 70 | 250 | 60 | 220 | 140 | 350 | 100 | 280 | 130 | 375 | 90 | 270 | 102 | 305 | 85 | 240 | 90 | 243 | 90 | 244 |
|  | Other leisure | 70 | 160 | 80 | 170 | 90 | 305 | 90 | 310 | 70 | 240 | 80 | 260 | 60 | 288 | 70 | 330 | 105 | 305 | 85 | 320 |
| 18-29 | Sleeping | 410 | 540 | 420 | 550 | 450 | 640 | 470 | 620 | 460 | 620 | 460 | 630 | 435 | 600 | 450 | 600 | 445 | 648 | 474 | 640 |
|  | Personal care | 50 | 90 | 60 | 110 | 20 | 60 | 30 | 80 | 30 | 70 | 40 | 90 | 20 | 55 | 30 | 70 | 25 | 60 | 30 | 80 |
|  | Meals | 30 | 80 | 30 | 80 | 40 | 90 | 40 | 90 | 40 | 110 | 40 | 100 | 30 | 75 | 30 | 80 | 25 | 70 | 30 | 70 |
|  | Paid work | 340 | 520 | 310 | 490 | 330 | 520 | 300 | 470 | 400 | 560 | 340 | 520 | 415 | 550 | 385 | 520 | 350 | 532 | 284 | 525 |
|  | Study | 180 | 470 | 120 | 430 | 110 | 370 | 100 | 335 | 170 | 430 | 120 | 390 | 180 | 491 | 150 | 455 | 120 | 395 | 94 | 338 |
|  | Housework | 20 | 80 | 40 | 160 | 40 | 130 | 50 | 160 | 30 | 120 | 50 | 200 | 30 | 130 | 55 | 203 | 20 | 135 | 50 | 195 |
|  | Care for others | 20 | 80 | 60 | 260 | 40 | 180 | 60 | 320 | 30 | 120 | 70 | 230 | 30 | 120 | 90 | 305 | 10 | 120 | 35 | 197 |
|  | Active leisure | 40 | 110 | 40 | 90 | 50 | 130 | 40 | 110 | 50 | 120 | 40 | 100 | 60 | 170 | 50 | 120 | 60 | 180 | 25 | 108 |
|  | Screen-based leisure | 90 | 280 | 70 | 210 | 110 | 350 | 80 | 240 | 100 | 310 | 80 | 240 | 90 | 270 | 60 | 210 | 90 | 285 | 90 | 240 |
|  | Other leisure | 100 | 230 | 90 | 230 | 60 | 280 | 60 | 250 | 50 | 210 | 60 | 230 | 60 | 328 | 67 | 285 | 60 | 330 | 60 | 270 |
| 30-44 | Sleeping | 420 | 530 | 410 | 520 | 440 | 580 | 450 | 580 | 440 | 580 | 450 | 580 | 420 | 540 | 435 | 555 | 425 | 585 | 450 | 600 |
|  | Personal care | 50 | 80 | 40 | 90 | 20 | 60 | 30 | 80 | 30 | 60 | 40 | 80 | 20 | 45 | 30 | 60 | 30 | 60 | 30 | 75 |
|  | Meals | 40 | 90 | 50 | 100 | 50 | 100 | 50 | 100 | 40 | 100 | 40 | 110 | 30 | 90 | 30 | 85 | 30 | 80 | 30 | 75 |
|  | Paid work | 370 | 550 | 290 | 480 | 390 | 530 | 360 | 490 | 400 | 560 | 250 | 490 | 430 | 585 | 375 | 523 | 360 | 590 | 245 | 513 |
|  | Study | 60 | 240 | 60 | 200 | 130 | 360 | 90 | 290 | 70 | 260 | 55 | 155 | 110 | 450 | 60 | 305 | 118 | 344 | 90 | 295 |
|  | Housework | 20 | 90 | 110 | 280 | 40 | 160 | 100 | 250 | 30 | 160 | 90 | 270 | 45 | 185 | 80 | 276 | 30 | 160 | 65 | 255 |
|  | Care for others | 30 | 90 | 30 | 180 | 30 | 130 | 30 | 170 | 30 | 140 | 50 | 190 | 45 | 150 | 60 | 213 | 30 | 152 | 45 | 195 |
|  | Active leisure | 40 | 110 | 50 | 100 | 40 | 140 | 40 | 100 | 50 | 120 | 40 | 110 | 60 | 149 | 35 | 105 | 30 | 160 | 27 | 90 |
|  | Screen-based leisure | 70 | 210 | 60 | 180 | 90 | 250 | 60 | 190 | 90 | 260 | 70 | 200 | 65 | 220 | 60 | 180 | 90 | 270 | 75 | 230 |
|  | Other leisure | 90 | 210 | 80 | 190 | 50 | 220 | 60 | 220 | 40 | 190 | 50 | 200 | 55 | 250 | 60 | 246 | 60 | 240 | 60 | 237 |
| 45-64 | Sleeping | 410 | 510 | 410 | 510 | 440 | 570 | 450 | 570 | 420 | 560 | 450 | 570 | 420 | 540 | 430 | 565 | 425 | 597 | 450 | 600 |
|  | Personal care | 50 | 90 | 50 | 90 | 20 | 60 | 30 | 70 | 30 | 70 | 40 | 80 | 20 | 55 | 30 | 65 | 30 | 60 | 30 | 80 |
|  | Meals | 40 | 100 | 50 | 100 | 50 | 100 | 50 | 110 | 50 | 120 | 50 | 120 | 35 | 95 | 30 | 90 | 30 | 80 | 30 | 75 |
|  | Paid work | 330 | 530 | 250 | 500 | 390 | 520 | 360 | 490 | 360 | 550 | 270 | 500 | 415 | 575 | 375 | 525 | 330 | 580 | 285 | 530 |
|  | Study | 50 | 180 | 60 | 150 | 70 | 280 | 75 | 250 | 50 | 150 | 30 | 150 | 60 | 260 | 60 | 195 | 90 | 450 | 90 | 255 |
|  | Housework | 20 | 100 | 120 | 280 | 60 | 210 | 100 | 260 | 50 | 200 | 110 | 290 | 50 | 225 | 77 | 285 | 30 | 180 | 60 | 260 |
|  | Care for others | 10 | 60 | 20 | 70 | 20 | 100 | 10 | 80 | 20 | 80 | 20 | 70 | 30 | 150 | 40 | 180 | 15 | 105 | 16 | 123 |
|  | Active leisure | 50 | 140 | 50 | 120 | 50 | 150 | 50 | 130 | 40 | 130 | 40 | 120 | 45 | 150 | 40 | 120 | 30 | 180 | 25 | 105 |
|  | Screen-based leisure | 80 | 230 | 70 | 200 | 110 | 290 | 70 | 210 | 110 | 310 | 90 | 230 | 95 | 270 | 75 | 240 | 115 | 350 | 90 | 280 |
|  | Other leisure | 100 | 230 | 80 | 220 | 70 | 250 | 80 | 270 | 60 | 230 | 60 | 230 | 61 | 276 | 80 | 303 | 60 | 240 | 73 | 270 |
| 65+ | Sleeping | 440 | 560 | 450 | 570 | 470 | 590 | 490 | 590 | 450 | 560 | 450 | 570 | 450 | 570 | 470 | 590 | 475 | 600 | 475 | 620 |
|  | Personal care | 50 | 90 | 40 | 90 | 30 | 80 | 30 | 80 | 30 | 80 | 40 | 90 | 20 | 60 | 30 | 70 | 30 | 60 | 30 | 90 |
|  | Meals | 60 | 110 | 60 | 100 | 70 | 120 | 60 | 120 | 70 | 150 | 70 | 150 | 45 | 120 | 45 | 110 | 30 | 95 | 30 | 90 |
|  | Paid work | 130 | 460 | 70 | 410 | 80 | 360 | 105 | 340 | 110 | 450 | 80 | 320 | 184 | 510 | 165 | 468 | 180 | 508 | 165 | 505 |
|  | Study | 60 | 160 | 60 | 150 | 40 | 220 | 90 | 140 | 50 | 160 | 30 | 130 | 60 | 187 | 90 | 140 | 45 | 248 | 60 | 95 |
|  | Housework | 30 | 120 | 120 | 260 | 70 | 230 | 130 | 300 | 80 | 220 | 140 | 300 | 60 | 265 | 95 | 290 | 33 | 180 | 65 | 260 |
|  | Care for others | 20 | 90 | 20 | 100 | 10 | 60 | 20 | 120 | 10 | 70 | 20 | 70 | 45 | 230 | 30 | 147 | 10 | 105 | 10 | 108 |
|  | Active leisure | 60 | 160 | 50 | 120 | 50 | 150 | 30 | 110 | 40 | 150 | 40 | 110 | 45 | 165 | 30 | 120 | 30 | 150 | 20 | 96 |
|  | Screen-based leisure | 130 | 330 | 110 | 280 | 130 | 300 | 110 | 250 | 140 | 350 | 120 | 270 | 140 | 360 | 120 | 320 | 165 | 470 | 130 | 375 |
|  | Other leisure | 100 | 270 | 100 | 270 | 120 | 340 | 140 | 340 | 90 | 270 | 110 | 290 | 120 | 390 | 155 | 410 | 119 | 353 | 120 | 360 |

Source: Multinational Time Use Study [32].

Figure A1. Gender Differences in Time Use with Three employment status types: employed, unemployed, retired

**
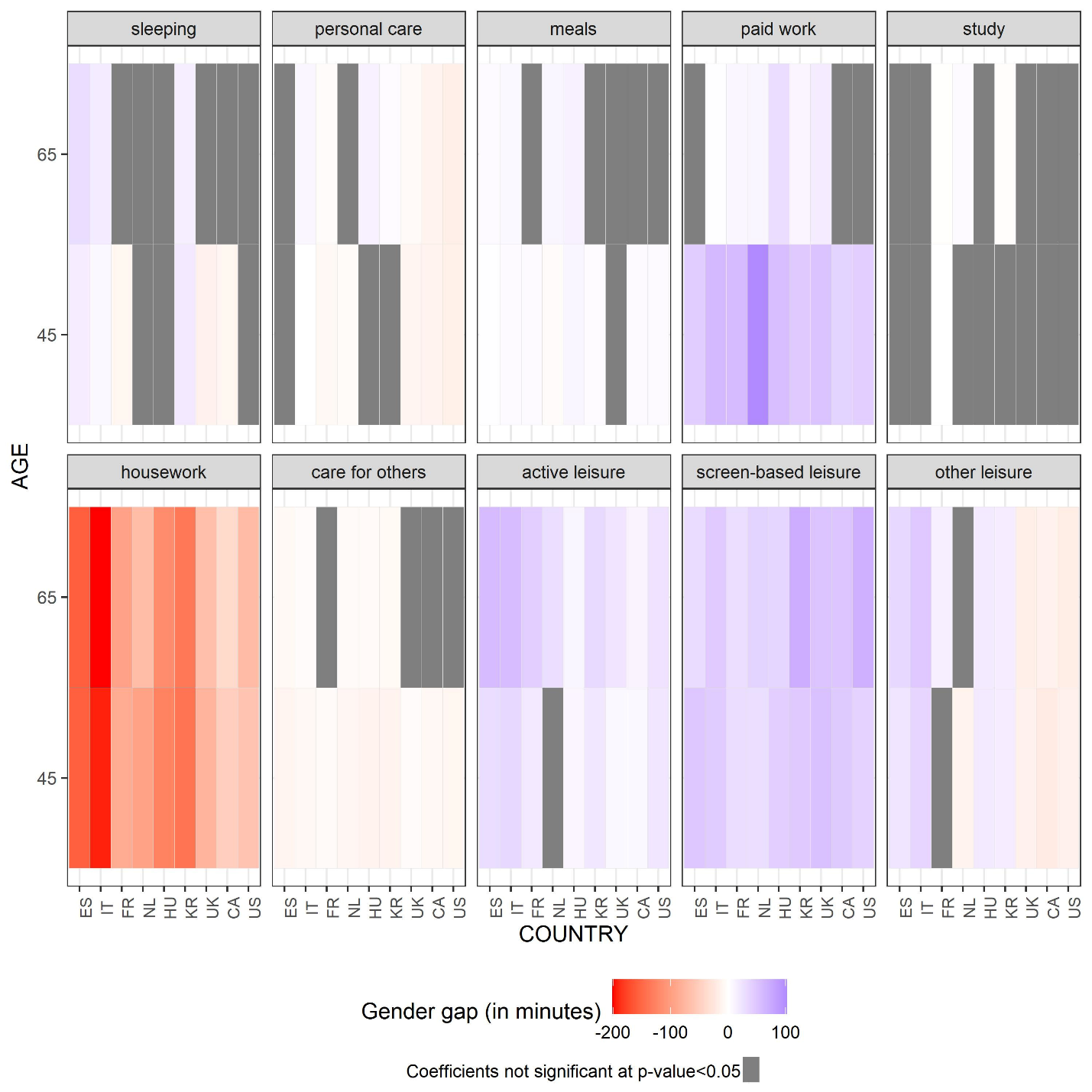
**

Source: Own calculations from the Multinational Time Use Study [32].

Notes: Estimates correspond to the coefficient for Category Men (ref=Women) from the OLS regressions by country and age groups, including the groups 45-64 and 65 and older. All regressions control by day of the week, educational attainment, employment status in 3 categories, children in the household, and union status. For Finland it is not possible to separate retired and not employed with the data.
